# 3D hydrogen-like screening effect on excitons in hBN-encapsulated monolayer transition metal dichalcogenides

**DOI:** 10.1038/s41598-024-77625-x

**Published:** 2024-11-08

**Authors:** S. Takahashi, S. Kusaba, K. Watanabe, T. Taniguchi, K. Yanagi, K. Tanaka

**Affiliations:** 1 Department of Physics, Kyoto University, Kitashirakawa-Oiwake-cho, Sakyo-ku, Kyoto, 606-8502 Japan; 2 Research Center for Electronic and Optical Materials, National Institute for Materials Science, 1-1 Namiki, Tsukuba, 305-0044 Japan; 3 Research Center for Materials Nanoarchitectonics, National Institute for Materials Science, 1-1 Namiki, Tsukuba, 305-0044 Japan; 4 Department of Physics, Tokyo Metropolitan University, 1-1 Minami-Osawa, Hachioji, Tokyo 192-0397 Japan; 5 Institute for Integrated Cell-Material Sciences, Kyoto University, Yoshida-Ushinomiya-cho, Sakyo-ku, Kyoto, 606-8502 Japan

**Keywords:** Optics and photonics, Physics

## Abstract

We observe both s-series and p-series excitons by using sum frequency generation spectroscopy on monolayer (1L-)MX_2_ (M = Mo, W, X = S, Se) encapsulated by hexagonal boron nitride (hBN). Moreover, we perform numerical calculations with the Rytova-Keldysh potential and obtain the relative dielectric constant of hBN among other parameters. The obtained relative dielectric constant can be approximated by the high-frequency limit of the infrared dispersion even though the exciton binding energies are almost on the phonon resonances in hBN. This suggests that the theoretically indicated modification of the exciton level structure due to the phonon resonances is negligible. The power-law scaling of exciton binding energies indicates that dielectric screening of 1L-MX_2_ exciton levels other than 1s can be approximated by that of a 3D hydrogen model with the dielectric constant of hBN.

**Supplementary Information:**

The online version contains supplementary material available at 10.1038/s41598-024-77625-x.

## Introduction

The characteristics of the Coulomb interaction in low-dimensional systems are different from those in three-dimensional systems^1–6^. One instance is that the Coulomb potential is modulated by the dielectric constant of the surroundings. In particular, the modified potential in a thin film has been approximated by the Rytova-Keldysh potential (RKP)^1,2^. Excitons, i.e., bound electron-hole pairs, are excellent platforms for studying such non-conventional Coulomb interactions in low-dimensional systems^3–6^ since their detailed bound state structure can be resolved by various optical spectroscopies.

Monolayer transition metal dichalcogenides (1L-TMDs) have emerged as playgrounds for studying exciton physics in two dimensions^7,8^. 1L-TMDs are direct-gap semiconductors with hexagonal crystal planes and are only three atomic thicknesses^9–12^. Because of the modified Coulomb potential, excitons in 1L-TMDs have binding energies of up to a few hundred meV and non-hydrogenic energy level structures^13–20^.

Exciton levels with large radius are useful for investigating the effect of dielectric screening in the surroundings. This is because larger the distance between charged particles in a thin film is, more lines of electric force leak outside the film. Observation of such high-order exciton levels has been enabled by a recently emerging encapsulation technique that uses hexagonal boron nitride (hBN); this technique allows clear exciton spectral lines in 1L-TMDs^15–24^ to be obtained by suppressing inhomogeneous broadening: hBN is a van-der-Waals layered insulator that is transparent in the visible region. The hBN layers on the bottom provides an atomically flat surface for 1L-TMDs and the hBN on the top protects 1L-TMDs from gas adsorption.

Previous studies^15,18^ used this technique to observe s-series excitons with a principal quantum number of $n$=3, 4, or 5 depending on material in hBN-encapsulated 1L-TMDs. They showed that numerical calculations with the RKP well reproduced the energy level structure of excitons in five hBN-encapsulated 1L-TMDs (MoS_2_, WS_2_, MoSe_2_, WSe_2_, and MoTe_2_). They used these calculations to obtain excitonic parameters, including the dielectric constant of hBN. The obtained values for this constant were almost the same for the five 1L-TMDs and consistent with the high-frequency limit of the infrared dispersion in an experimental report^25^. On the other hand, it was theoretically proposed that the phonon resonances of the surrounding hBN layers significantly modify the exciton energy structure in 1L-TMDs^26^.

The effects of phonon resonances have also been discussed for II-VI semiconductors^27,28^ and halide perovskites^29–32^. In these materials, LO phonons have frequencies comparable with exciton binding energies. This results in large difference of the static and optical dielectric constants. It is theoretically unknown what value of dielectric constant should be taken to describe exciton binding energy. In addition, it is also hard to directly measure exciton binding energy only by experiments. Therefore, both the dielectric constant and the exciton binding energy are uncertain and difficult to determine independently. Here, we address this issue through a combination of nonlinear spectroscopy and phenomenological analysis.

The effect of the dielectric constant of the surroundings can be probed more clearly by simultaneously measuring s-series and p-series exciton energies. In contrast to 3D bulk semiconductors, the degeneracy between the s and p levels with the same principal quantum number is lifted in 1L-TMDs because of the modified Coulomb potential^20,33,34^. P-series excitons can only be observed with nonlinear spectroscopies^20,23,33,35^; consequently, they have been observed in different experiments from those for observing s-series excitons. On the other hand, it is well known that exciton energies fluctuate even within the same sample^36^. Therefore, the 1L-TMD exciton level structure should be determined on the same sample spot by using the same experimental technique. Recently, we simultaneously observed s-series and p-series excitons in hBN-encapsulated 1L-WSe_2_ by using sum frequency generation (SFG) spectroscopy^20^.

In this paper, we describe an SFG spectroscopy observation of s-series and p-series excitons on four 1L-TMDs (MoS_2_, WS_2_, MoSe_2_, and WSe_2_) encapsulated by hBN and discuss the exciton level structure by using numerical calculations with the RKP. We find that the calculations with the RKP reproduce exciton energies including the s-series and p-series levels obtained by SFG spectroscopies to within a few meV. Moreover, the relative dielectric constants of hBN for the four 1L-TMDs can be approximated by the high frequency limit of the infrared dispersion, similarly to the previous studies^15,18^, even though the exciton binding energies, especially those of 1s levels, are almost on the phonon resonances. Furthermore, we obtain a power-law scaling of exciton binding energies that is similar to that for the 3D hydrogen model, indicating that dielectric screening of excitons other than 1s can be approximated by that of the 3D hydrogen model with the dielectric constant of hBN.

## Results

### Experiments

**Fig. 1:**
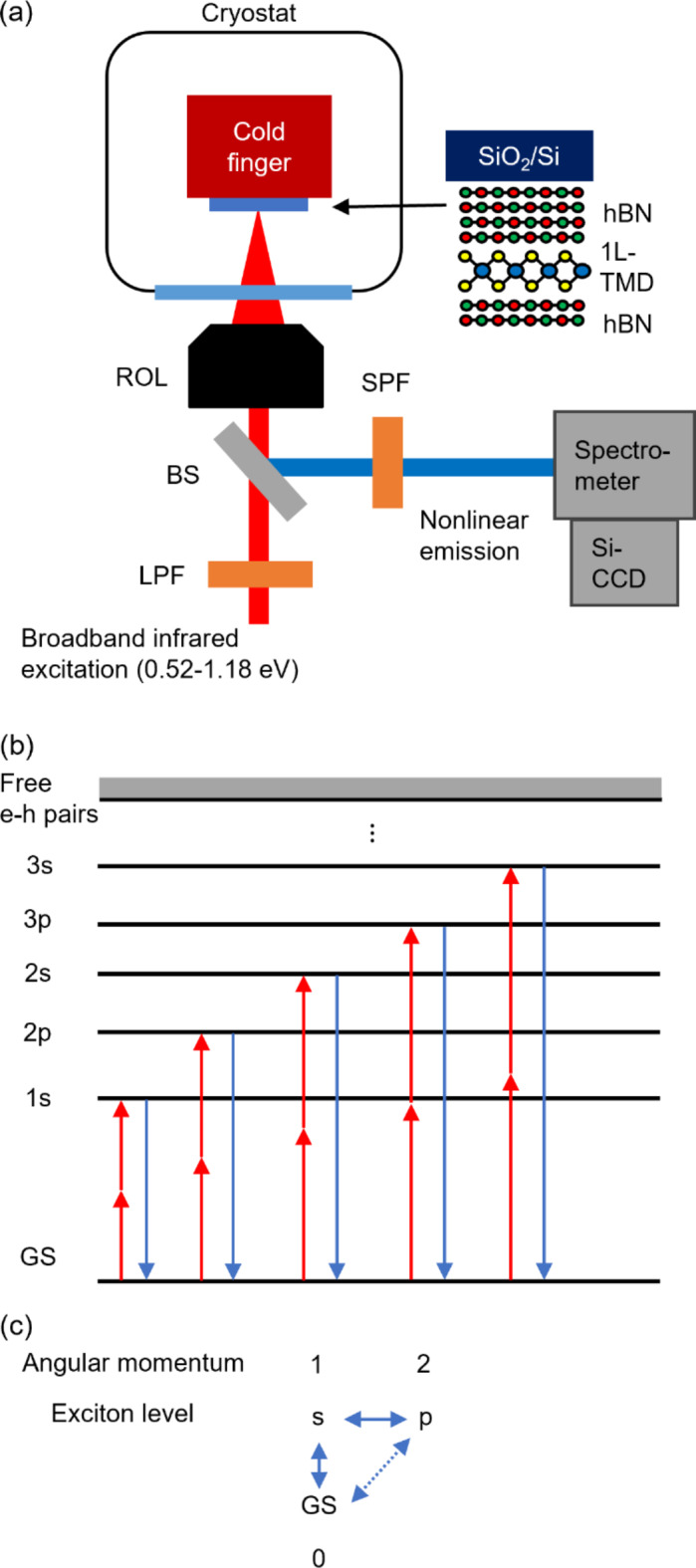
(**a**) Schematic setup of the experiment. Broadband near-infrared excitation light (0.52–1.18 eV) from a supercontinuum light source is focused on the hBN-encapsulated 1L-TMD sample in a cryostat by a reflection objective lens (ROL). Nonlinear emissions from the sample are collected by the same lens and detected by a silicon-based charge-coupled detector (Si-CCD) attached to a spectrometer. BS stands for beam splitter and LPF (SPF) long (short) pass filter. The thicknesses of bottom hBN are estimated by reflection measurements to be around 342, 350, and 220 nm for MoS_2_, MoSe_2_, and WS_2_ samples, respectively. The thicknesses of top hBN are around 8 nm for the three samples. (**b**) Representative energy diagram of SFG processes for 1L-TMD excitons. Red and blue arrows indicate excitations and emissions, respectively. GS is the ground state. (**c**) Optical selection rule of 1L-TMDs. The solid arrows show dipole-allowed transitions, and the dotted arrow shows a transition partially allowed by the threefold rotational symmetry of the 1L-TMD crystal.

hBN-encapsulated 1L-TMD samples are prepared on 300-nm SiO_2_/Si substrates by using mechanical exfoliation and dry transfer^37^. The thicknesses of bottom hBN are estimated by reflection measurements to be around 342, 350, and 220 nm for MoS_2_, MoSe_2_, and WS_2_ samples, respectively. The thicknesses of top hBN are around 8 nm for the three samples. We confirm that the samples are composed of 1L-TMDs and hBN having the good crystallinity by Raman spectra and intense PL at ambient conditions. Details on the preparation and characterization are described in section S1 and Figs. S1 in the supplementary information. Figure 1a shows a schematic diagram of the experimental setup for SFG spectroscopy. The method is basically the same as in our previous report^20^. Broadband near-infrared excitation light (0.52–1.18 eV) from a supercontinuum light source is focused on an hBN-encapsulated 1L-TMD sample in a cryostat by using a reflection objective lens. Nonlinear emissions from the sample are collected by the same lens, led to a spectrometer, and detected by a silicon-based charge-coupled detector.

This method enables us to measure nonlinear emissions resonant to exciton levels in 1L-TMDs by taking single scans for the following reasons. The excitation light has a smooth and broadband spectrum covering the range where resonant SFG processes of exciton levels occur in four 1L-TMDs. Due to chirping, photons with an energy difference of $\sim$60 meV were found to contribute to SFG^20^. Since the thicknesses of 1L-TMDs are much smaller than the wavelengths of the excitation and the emission, we do not need to consider the phase matching conditions for SFG processes. The use of reflection objective lens allows us to ignore chromatic aberration. That is why the resultant SFG spectrum is almost independent of the details of the excitation profile and the other measurement conditions^20,38^.

Figures 1b and c show the optical transitions and selection rule in 1L-TMDs, respectively. In linear optical processes, s-series excitons have much larger oscillator strengths than those of other series excitons, because the oscillator strengths are proportional to $\:{\left|{\Psi}\left(\overrightarrow{r}=0\right)\right|}^{2}$, where $\:{\Psi}\left(\overrightarrow{r}\right)$ is the exciton orbital function^34^. Therefore, linear spectroscopies can only observe s-series excitons. On the other hand, in SFG processes, both s-series and p-series levels are observable as shown in Figs. 1b, c because of the trigonal warping effect of electronic bands^39,40^. SFG spectroscopy is thus useful in that it allows us to determine both s-series and p-series exciton levels in a single measurement.

**Fig. 2:**
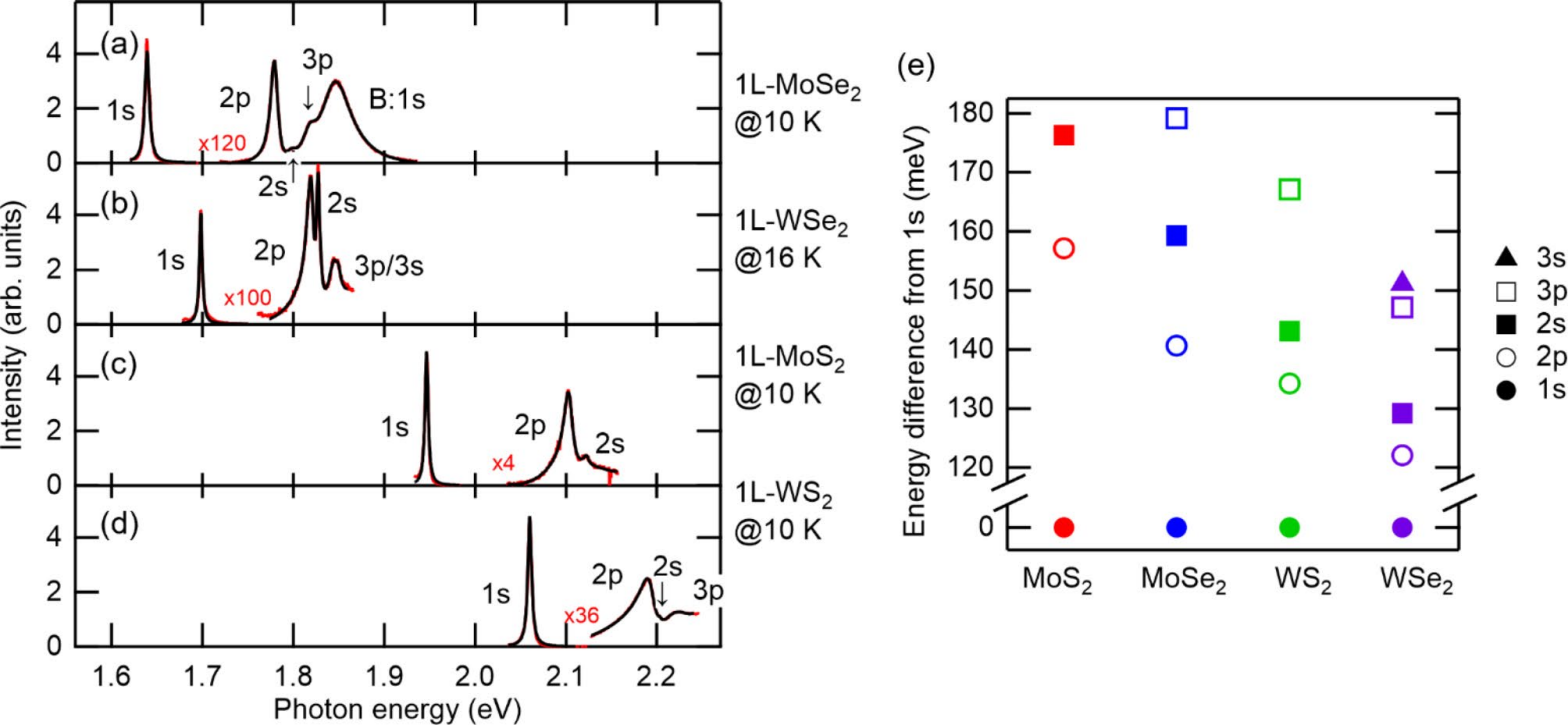
Nonlinear emission spectra for (**a**) 1L-MoSe_2_, (**b**) 1L-WSe_2_, (**c**) 1L-MoS_2_, and (**d**) 1L-WS_2_. Red and black lines are experimental data and fitting curves (described in the text), respectively. The data for 1L-WSe_2_ are taken from our previous report^20^. (**e**) Exciton energy level structures in four 1L-TMDs. Energy differences from 1s levels are plotted.

Representative nonlinear emission spectra for four 1L-TMDs taken at low temperature are shown in Figs. 2a–d. The data for 1L-WSe_2_ in Fig. 2b are from our previous report^20^. hBN encapsulation enables us to observe clear peak structures. The origins of the observed peaks can be assigned by comparing the spectra with those observed by linear spectroscopies (see section S2 and Figs. S2-S4). For instance, in 1L-MoSe_2_ in Fig. 2a, we observe 1s, 2p, 2s, 3p, and B:1s excitons. The B:1s exciton originates from the spin-orbit split band of a hole. 1s, 2p, and 2s excitons are similarly observable in other 1L-TMDs, and depending on material, 3p and 3s exciton lines can also be seen. The weak and broad B:1s exciton line, which is resolved only in the spectral fitting described below, overlaps those of the 2p and 2s excitons in 1L-MoS_2_. In W-based 1L-TMDs, the energy difference between the B:1s and 1s exciton level is larger than those in Mo-based 1L-TMDs because of the relatively large spin-orbit splitting and the signals from the B:1s excitons are too weak to observe. In the nonlinear emission spectra, we also observe trions as shown in Figs. S2-S4 in the supplementary information. However, densities of unintentionally doped carriers are small (< 10^9^ cm^− 2^) enough to neglect their effect such as their screening effect and exciton energy shift^41^, which are estimated from the ratio of resonant SFG components of the 1s exciton and trion lines. Details are described in section S3 in the supplementary information.

In the spectra in Figs. 2a–d from the four 1L-TMDs, the 2p excitons have larger linewidths than those of 1s excitons. This is ascribed to the 2p excitons having more decay channels than the 1s excitons, such as 1s and dark exciton levels. We can also see that the nonlinear emissions of the 2p exciton levels are weaker than those of the 1s exciton levels. This can be explained as follows. Firstly, the 1s exciton lines originate from both the resonant SFG processes and two-photon photoluminescence (2P-PL), while the 2p exciton lines mostly originate from resonant SFG processes. 2P-PL is where excitation to energetically higher states by two photons, non-radiative relaxation to lower states, and luminescence occur in this order. Secondly, the 2p excitons have larger linewidths $\gamma$, resulting in smaller peak heights which scale as $1/\gamma$.

In all of the materials, the p levels are lower in energy than s levels having the same principal quantum number. This result is consistent with those in previous studies^33,34^ and is ascribed to orbital-dependent dielectric screening effects. The spectra are arranged in order of band-gap energy. We can see that the type of chalcogen mainly determines the size of the band gap and the type of transition metal has a secondary effect.

To determine the exciton energies, the spectral peaks can be fitted using the following function:

1 $$\begin{aligned}S\left(\omega\right)&=\left(\text{c}\text{o}\text{h}\text{e}\text{r}\text{e}\text{n}\text{t}\:\text{p}\text{a}\text{r}\text{t}\right)+\left(\text{i}\text{n}\text{c}\text{o}\text{h}\text{e}\text{r}\text{e}\text{n}\text{t}\:\text{p}\text{a}\text{r}\text{t}\right) \\  & { = \left| {a + b\hbar \omega  + \sum\limits_{j} {\dfrac{{\sqrt {A_{j} } }}{{\hbar \omega  - \epsilon _{j} + i{\gamma _{j} / 2}}}} } \right|^{2} + \sum\limits_{j} {\dfrac{{B_{j} }}{{\left( {\hbar \omega  - \epsilon _{j} } \right)^{2} + \left( {\gamma _{j} / 2} \right)^{2} }}} ,} \\   \end{aligned}$$

where $\hslash\omega$ is photon energy, and ${\epsilon}_{j}$,  ${\gamma}_{j}$, and ${A}_{j}$ (${B}_{j}$) are exciton energy, full width at half maximum (FWHM), and peak intensity of the $j$-th exciton level, respectively. The coherent part corresponds to resonant SFG. The linear term in the coherent part is the non-resonant component. The incoherent part in Eq. (1) originates from 2P-PL. Contributions from resonant SFG and 2P-PL can be distinguished by their polarization dependences: the former obeys the selection rule of the second-order nonlinearity in 1L-TMDs whereas the latter has a polarization parallel to the excitation polarization or loses information on it. The best fitted curves to the data (fitting parameters are ${\epsilon}_{j}$, ${\gamma}_{j}$, ${A}_{j}$, and (${B}_{j}$) are shown by the black lines in Figs. 2a–d. The procedure for separating the incoherent part and performing the fitting are described in sections S2 and S4, Figs. S2-S5, and Tables S1-S5 (see also our previous report^20^). The fitting functions of the 1s peaks are the usual Lorentzian functions, because they are energetically apart from the other levels. The experimental data are well reproduced by Lorentzian functions, which indicates that the exciton linewidths are dominated by homogeneous broadening owing to the high quality of the hBN-encapsulated samples.

The obtained exciton energies are summarized in Table 1 and plotted in Fig. 2e as energy differences from the 1s level in each material. Materials are arranged in order of 1s-2p level splitting, which is different from the order of band-gap energy in Figs. 2a–d. In Fig. 2e, exciton level structure seems to depend mainly on the type of transition metal, and secondarily on that of chalcogen. Note that we cannot determine the binding energies or band-gap energies from the experimental data, but we can determine energy separations from the 1s exciton level.

**Table 1:** Exciton energies obtained by performing SFG spectroscopy on the four 1L-TMDs in Figs. 2a–d.

Orbital	Energy obtained by experiments (eV)			
	MoS_2_	MoSe_2_	WS_2_	WSe_2_
1s	1.9466$\pm$0.0006	1.6390$\pm$0.0006	2.0601$\pm$0.0006	1.6982$\pm$0.0006
2p	2.1038$\pm$0.0006	1.7797$\pm$0.0006	2.1944$\pm$0.0006	1.8203$\pm$0.0006
2s	2.1229$\pm$0.0007	1.7983$\pm$0.0006	2.2033$\pm$0.0006	1.8273$\pm$0.0006
3p		1.8182$\pm$0.0006	2.2272$\pm$0.0007	1.8453$\pm$0.0007
3s				1.8489$\pm$0.0008

The errors of the experimental values are determined by the spectral resolution ($\sim$0.6 meV) and the fitting error. The experimental data for 1L-WSe_2_ are from our previous report^20^.

### Numerical calculations

Here, we examine how well the RKP model reproduces the exciton energy level structure including both s-series and p-series levels. We neglect other contributions, such as Berry curvature effects^23,42–45^, as smaller ones. The RKP is effectively expressed as^1,2^

2 $$\begin{array}{c}{V}_{\text{R}\text{K}}\left(r\right)=-\dfrac{{e}^{2}}{8{\varepsilon}_{0}{r}_{0}}\left[{H}_{0}\left(\dfrac{\kappa r}{{r}_{0}}\right)-{Y}_{0}\left(\dfrac{\kappa r}{{r}_{0}}\right)\right],\end{array}$$

where $e$ and ${\varepsilon}_{0}$ are the elementary charge and permittivity in vacuum, respectively, and ${H}_{0}$ and ${Y}_{0}$ are the Struve function and Bessel function of the second kind, respectively. The use of the RKP is justified by that the effective thicknesses of 1L-TMDs^46^ of $\sim$0.1 nm is smaller than the estimated exciton radii^47^ of $\gtrsim$1 nm. We hereby assume that the dielectric parameters of hBN and 1L-TMDs can be described by certain effective values. We use the exciton reduced masses determined in previous studies^15,18^. We numerically calculate exciton binding energies with two adjusting parameters, i.e., the relative dielectric constant of hBN $\kappa$ and the screening length of 1 L-TMD ${r}_{0}$. We define deviation of the numerically calculated binding energy separations from those in the experiments by

3 $$\begin{array}{*{20}c} {\sigma = \sqrt {\mathop \sum \limits_{{j = 2p,2s, \ldots }} \left( {\Delta \epsilon _{\mathrm{calc}}^{j} - \Delta \epsilon _{\mathrm{exp }}^{j} } \right)^{2} } ,} \\ \end{array}$$

where ${\Delta}{\epsilon}^{j}$ is the energy difference between 1s and another level $j=2p,2s,\dots$ in the calculation or experiment. The parameters $\kappa$ and ${r}_{0}$ for each material are those that minimize $\sigma$. By using these adjusted parameters, we can determine the band-gap energy ${E}_{g}$ from the sum of the numerically obtained 1s exciton binding energy and the experimentally obtained 1s exciton energy. The adjusted parameters are summarized in Table 2.

**Table 2:** Parameters obtained in the numerical calculations.

Material	$\kappa$	${r}_{0}$ (nm)	$\left|{E}_{b}^{1s}\right|$ (meV)	${E}_{g}$ (eV)
MoS_2_	3.80$\pm$0.64	3.85$\pm$0.65	239.5$\pm$0.6	2.1861$\pm$0.0006
MoSe_2_	5.05$\pm$1.23	3.82$\pm$1.15	207$\pm$3	1.849$\pm$0.003
WS_2_	4.07$\pm$0.72	3.60$\pm$0.93	189$\pm$3	2.249$\pm$0.003
WSe_2_	5.01$\pm$0.66	3.45$\pm$0.82	164$\pm1$	1.862$\pm$0.001

The parameters are those that minimize the root-mean-square deviations of the numerically calculated exciton energies from the experimentally obtained exciton energies. $\kappa$ is the relative dielectric constant of hBN, ${r}_{0}$ the screening length of 1L-TMD, ${E}_{b}^{1s}$ the numerically calculated 1s exciton binding energy, and ${E}_{g}$ the band-gap energy.

We compare obtained band-gap energies and 1s exciton binding energies with those reported in previous papers^15–17,19^. They are found to disagree with each other with differences larger than the errors as shown in Table S6 in the supplementary information. This is possibly due to the difference of the dielectric environment^36^ such as the distance between hBN and 1L-TMD as described in section S6 in the supplementary information. We confirm that these differences do not influence our conclusion described below including power-law scaling in Fig. 4, as described in detail in section S9 in the supplementary information.

**Fig. 3:**
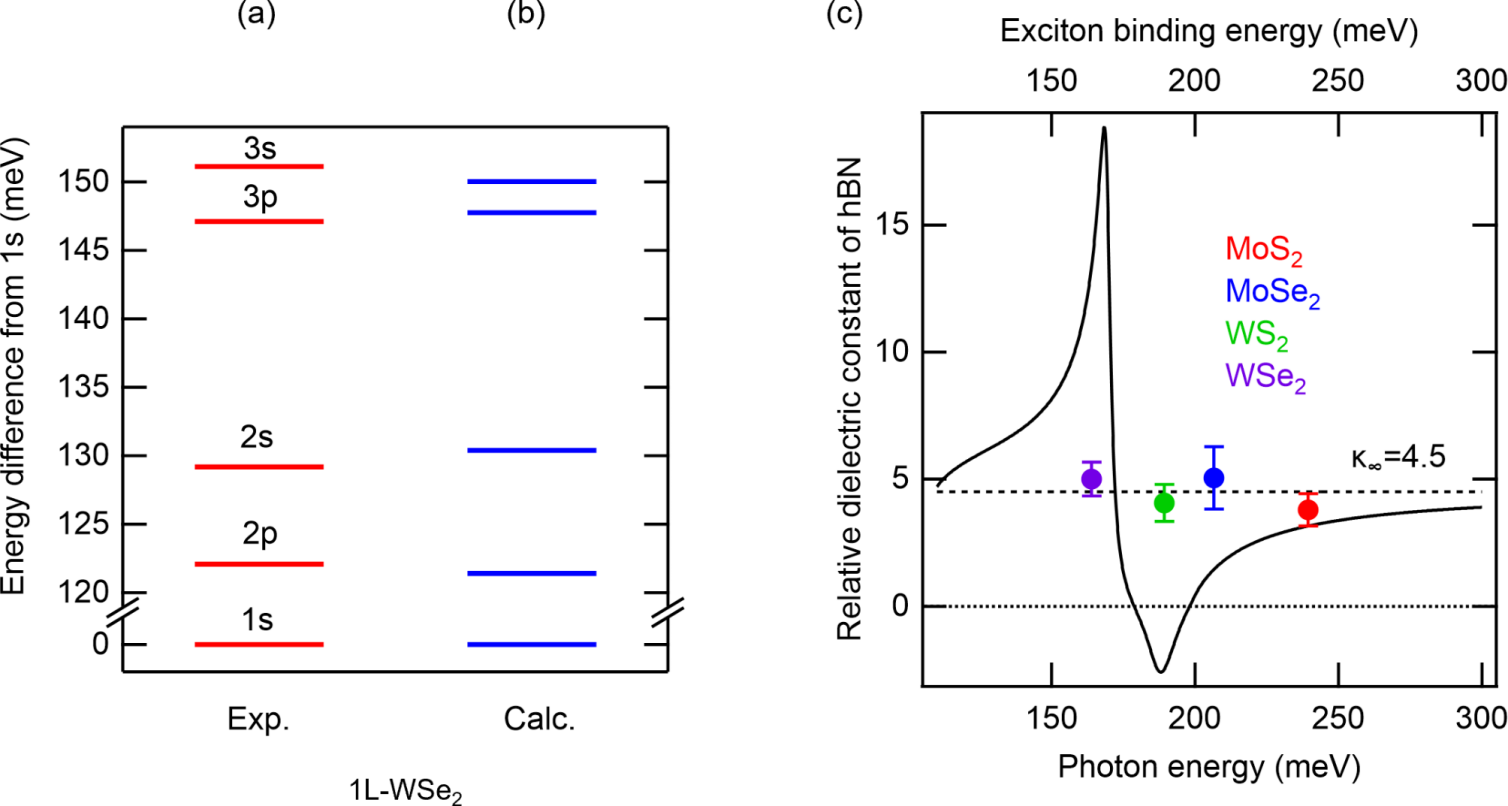
Comparison of exciton energy levels in (**a**) experiment and (**b**) numerical calculation for 1L-WSe_2_ as a representative. Energy differences from the 1s level are plotted. (**c**) The plots are 1s exciton binding energies (upper horizontal axis) plotted against the relative dielectric constant of hBN for four 1L-TMDs. The solid line is the energy dependence (lower horizontal axis) of the effective relative dielectric constant of hBN calculated using expression in ref.^48^ and the parameter values in ref.^25^. The errors on the vertical axis are the same as those tabulated in Table 2 and those on the horizontal axis are within the sizes of the plots.

The exciton energy level structures in the experiments and numerical calculations are compared in Table 3; the upper row lists binding energies obtained by taking the difference between exciton energies found in the experiments in Table 1 and the band-gap energy in Table 2, and the lower row gives the numerically calculated values by using the adjusted parameters $\kappa$ and ${r}_{0}$ in Table 2. Figures 3a and b show differences in energy from that of the 1s exciton for 1L-WSe_2_ as a representative obtained in the experiment and from calculations with the adjusted parameters, respectively. They almost coincide, with a difference of $\sim$1 meV. From this difference, we determine the errors of parameters in Table 2 in the manner described in section S5 in the supplementary information.

**Table 3:** Absolute values of binding energies for exciton levels in the four 1L-TMDs observed in the nonlinear emission spectra in Figs. 2a-d.

Orbital	Absolute value of binding energy (meV)			
	MoS_2_	MoSe_2_	WS_2_	WSe_2_
1s	239.5	207	187	164
	239.5	207	187	164
2p	82.3	66	52	42
	82.3	65	53	42
2s	63.2	47	44	35
	63.2	50	41	33
3p		27	20	17
		26	20	16
3s				13
				14

The upper rows are obtained by taking the difference between the exciton energies of the experiments in Table 1 and the band-gap energy ${E}_{g}$ in Table 2. The lower rows are the results of numerical calculations with the RKP model using the parameters $\kappa$ and ${r}_{0}$ in Table 2. The errors are 0.6, 3, 3, and 1 meV for MoS_2_, MoSe_2_, WS_2_, and WSe_2_, respectively.

One can see that, independent of materials, the adjusted parameter $\kappa$ is almost 4.5 within the errors, which corresponds to the high-frequency limit of the effective relative dielectric constant of hBN reported in previous studies^15,18^. As described in the introduction, hBN has phonon resonances near the exciton binding energies and it was theoretically reported to modify significantly the exciton level structure^26^. To see the situation, the 1s exciton binding energy $\left|{E}_{b}^{1s}\right|$ and adjusted $\kappa$ for the four 1L-TMDs are plotted in Fig. 3c. In addition, for comparison, the figure also plots the energy dependence of the effective relative dielectric constant of hBN, as calculated by the expression^48^ $\sqrt{{\varepsilon}_{\parallel}{\varepsilon}_{\perp}}$, where ${\varepsilon}_{\parallel}$ and ${\varepsilon}_{\perp}$ are the relative dielectric constants for light polarizations parallel and perpendicular to the c-axis of hBN, respectively^25^. It can be seen that the effective relative dielectric constants at 1s exciton binding energies $\left|{E}_{b}^{1s}\right|$ vary widely from material to material, whereas the adjusted $\kappa$ stays almost constant. This suggests that contributions to the relative dielectric constant of hBN for excitons in 1L-TMDs come exclusively from electronic transitions (${\kappa}_{\infty}$) and little from phonon resonances. In other words, screening of excitons is not so significantly affected by phonon resonances in hBN and manifested only by electronic contribution. The conclusion of weak coupling between excitons in 1L-TMDs and phonons in hBN is supported by the fact that it has not observed the phonon mode of hBN so far in the resonant Raman scattering or the phonon side band for spectrum of van der Waals heterostructures comprised of hBN and 1L-TMD under resonant excitation to the 1s exciton^49–53^. In contrast, strong phonon sidebands are observed in II-VI semiconductors and perovskites, where phonon resonances have a non-negligible effect on exciton dielectric screening^29,54^.

### Power-law scaling

By using the adjusted parameters, we can examine the power-law scaling of the exciton binding energies in 1L-TMDs. In the previous study^6^, the authors theoretically investigated the dependence of the 1s exciton binding energy ${E}_{b}$ on the surrounding dielectric constant and tube diameter of carbon nanotubes. They found a simple power-law scaling $m{R}^{2}\left|{E}_{b}\right|\propto{\left(mR/\varepsilon\right)}^{\alpha}$, where $m$, $R$, and $\varepsilon$ are exciton reduced mass, exciton size, and dielectric constant of the surroundings, respectively. The exponent was found to be $\alpha=1.40$. In this study, we rewrite this relation as a power-law scaling $\left|{E}_{b}\right|/{E}_{k}\propto{\left({|E}_{c}|/{E}_{k}\right)}^{\alpha}$, where ${E}_{c}$ is Coulomb potential energy ($=-{e}^{2}/4\pi\varepsilon{R}$), and ${E}_{k}$ is kinetic energy ($={\hslash}^{2}/2m{R}^{2}$). Furthermore, we propose here a new power-law scaling that is applicable to other levels including 2p, 2s, and so on, as well as the 1s level, which is different from the power-law scaling mentioned above.

Our new power-law scaling is a kind of “virial theorem”. Let us start with the 3D hydrogen model, from which we can derive analytical expressions^55^

4 $$\begin{array}{c}{E}_{b}=-\dfrac{Ry}{{n}^{2}},\end{array}$$

5 $${E_{c} = - \frac{{e^{2} }}{{4\pi \varepsilon}}\left\langle {R^{{ - 1}} } \right\rangle _{n} = - 2\frac{{Ry}}{{n^{2} }},}$$

and

6 $$\begin{array}{*{20}c} {E_{k} = \dfrac{{\hbar^{2}}}{{2~m}}\left\langle {R^{{ - 1}} } \right\rangle _{n} ^{2} = \dfrac{{Ry}}{{n^{4} }},} \\ \end{array}$$

where $Ry=m{e}^{4}/32{\pi}^{2}{\varepsilon}^{2}{\hslash}^{2}$ is the Rydberg constant, and all the energies depend only on the principal quantum number $n$. A detailed description is given in section S8. Using Eqs. (4), (5), and (6), we can derive the power-law scaling as

7 $$\begin{array}{c}\dfrac{\left|{E}_{b}\right|}{{E}_{k}}=C{\left(\dfrac{\left|{E}_{c}\right|}{{E}_{k}}\right)}^{\beta},\end{array}$$

where $\beta=1$ and $C=0.5$. This scaling can be understood as a form of virial theorem^55^, which ensures that the expectation value of the kinetic energy $$\begin{array}{*{20}c} {\left\langle K \right\rangle = p\left\langle V \right\rangle /2} \\ \end{array}$$ when the potential is a centrifugal one of the form $V\left(r\right)\propto{r}^{p}$. This leads to ${E}_{b} = \left\langle V \right\rangle/2$ for the 3D hydrogen model with $p=-1$.

**Fig. 4:**
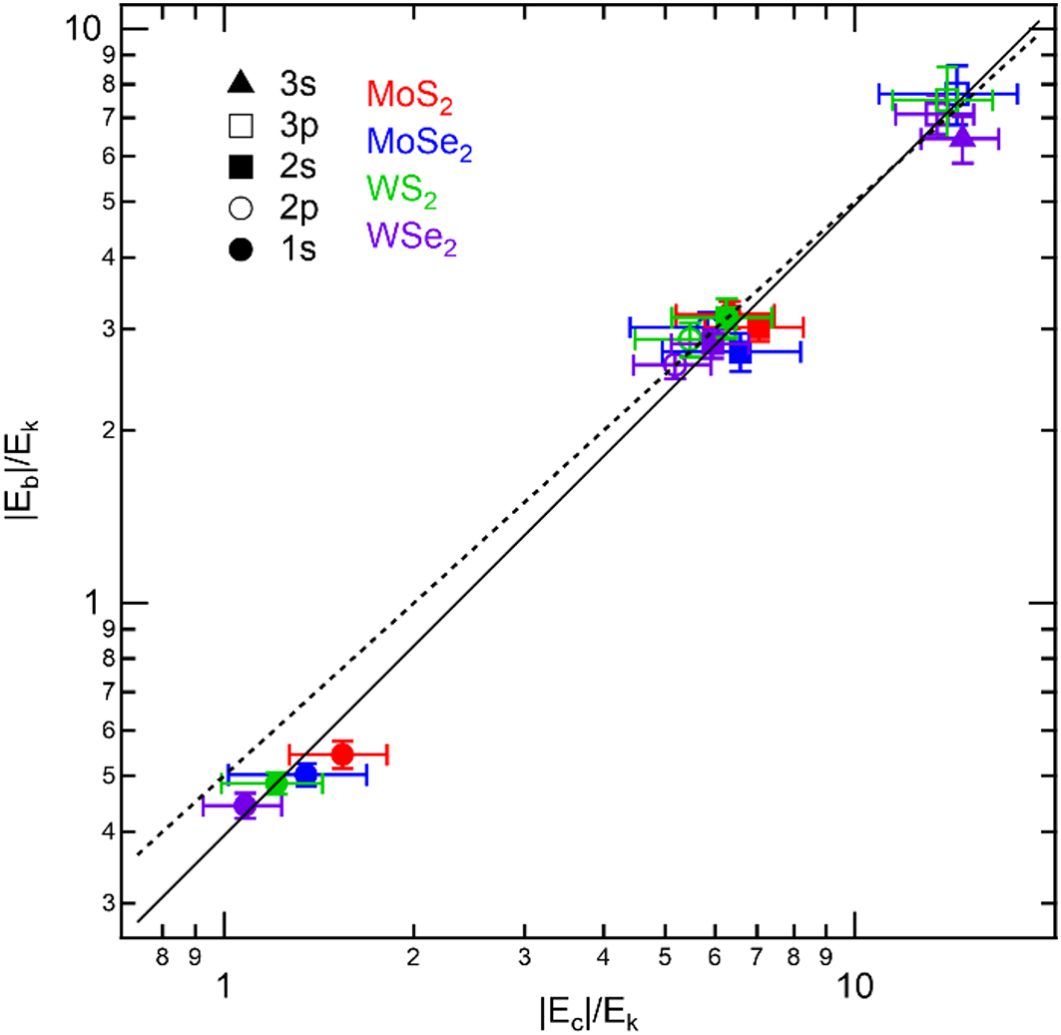
Absolute values of the scaled binding energy $\left|{E}_{b}\right|/{E}_{k}$ plotted against Coulomb potential energy $\left|{E}_{c}\right|/{E}_{k}$, where ${E}_{k}$ is kinetic energy. Plot shapes correspond to exciton levels and colors to materials and are the same as those in Fig. 2e. The solid line is the best fitted curve to Eq. (7) with $\beta=1.1\pm0.1$ and $C=0.4\pm0.1$, and the dashed line is the power-law scaling in the 3D hydrogen model with $\beta=1$ and $C=0.5$. The errors of the plots are estimated from those of the binding energies, relative dielectric constant of hBN, and exciton reduced mass.

Next, we calculate the power-law scaling for 1L-TMD excitons to examine how well dielectric screening can be described as that of the 3D hydrogen model. ${E}_{b}$ of each exciton level is calculated from the experimentally obtained exciton energy minus the numerically obtained band-gap energy ${E}_{g}$ listed in the upper rows in Table 3. ${E}_{c}$ and ${E}_{k}$ are calculated similarly to Eqs. (5) and (6):

8 $$\begin{array}{c}{E}_{c}=-\dfrac{{e}^{2}}{4\pi{\varepsilon}_{0}\kappa}{\left\langle{R}^{-1}\right\rangle}_{nl},\end{array}$$

and,

9 $$E_{k} = \dfrac{{\hbar^{2} }}{{2m}}\left\langle {R^{{ - 1}} } \right\rangle _{{nl}}^{2} ,$$

where ${\left\langle{R}^{-1}\right\rangle}_{nl}$ is the expected value of the reciprocal of the radius calculated using the exciton wave function with quantum numbers $n$ and $l$. The exciton wave functions are obtained from a numerical calculation of exciton binding energies with the adjusted parameters $\kappa$ and ${r}_{0}$ in Table 2. The details of the calculation are described in section S9. The exciton reduced mass $m$ is again taken from previous studies^15,18^. Figure 4 plots $\left|{E}_{b}\right|/{E}_{k}$ against $\left|{E}_{c}\right|/{E}_{k}$ for all exciton levels in the four 1L-TMDs observed in the nonlinear emission spectra in Figs. 2a–d. Here, we take the absolute values of ${E}_{b}$ and ${E}_{c}$ since they are negative. The order of the exciton levels is the opposite of that of ${E}_{b}$ because of ${E}_{k}$ in the denominator. Remarkably, the data for energetically separated 2p and 2s levels are at almost the same position in the graph. In addition, we can see that all the data are well reproduced by the best fitted curve to Eq. (7) with $\beta=1.1\pm0.1$ and $C=0.4\pm0.1$ (solid line). These values coincide with those for the 3D hydrogen model ($\beta=1,\:C=0.5$) within the errors.

The power-law scalings for the 3D hydrogen model and 1L-TMDs with the RKP are compared in Figs. S8. The data for exciton levels with $n=2,\:3$ are well reproduced by the power-law scaling for the 3D hydrogen model (dashed line). This behavior can be understood from that the RKP can be approximated for large exciton radius by usual Coulomb potential for the 3D hydrogen model (see Eq. (10) below and section S10 in the supplementary information). On the other hand, the data for the 1s levels in 1L-TMDs deviate slightly from the power-law scaling of the 3D hydrogen model. Moreover, the data for the 1s levels are distributed depending on the material. Within the 1s levels, we find another power-law scaling, with $\beta\sim0.6$. This power-law scaling may be ascribed to the deviation of the RKP from the usual Coulomb potential; the RKP in Eq. (2) can be approximated in the range $\kappa r/{r}_{0}\gg1$ as^1,2^

10 $$\begin{array}{c}{V}_{\text{R}\text{K}}\left(r\right)\sim-\dfrac{{e}^{2}}{4\pi{\varepsilon}_{0}\kappa r}.\end{array}$$

This is nothing but the 3D hydrogenic Coulomb potential. For the 1s exciton, $\kappa r/{r}_{0}$ is $\sim1$, so the effect of dielectric screening in a 1L-TMD should be significant (see section S10 in the supplementary information).

## Discussion

By using the RKP model, we find that the relative dielectric constant of hBN can be approximated by the high-frequency limit of the effective infrared dispersion for excitons in four 1L-TMDs. This result suggests that phonon resonances in hBN have negligible influence on the exciton level structure in 1L-TMDs. This conclusion is supported by the fact that it has not observed the phonon mode of hBN so far in the resonant Raman scattering or the phonon side band for spectrum of van der Waals heterostructures comprised of hBN and 1L-TMD under resonant excitation to the 1s exciton^49–53^. Our analyses have not considered the dispersion of dielectric constant. Our findings shall motivate further investigation of dynamical screening by phonons in hBN^26^, their polariton effects^56^ in the strong coupling regime, and substrates with large dielectric constant^57,58^.

The power-law scaling obtained from a combination of experiments and phenomenological analysis for the exciton binding energy in 1L-TMDs is almost the same as the one in the 3D hydrogen model, although it shows slight deviations for 1s excitons. This indicates that dielectric screening of exciton levels other than 1s can be approximated by that of the 3D hydrogen model with the dielectric constant of hBN. Dielectric screening of 1s excitons should include contributions from the 1L-TMDs, which results in a modified power-law scaling that only holds for the 1s level. The estimated exponent of this power-law scaling is $\sim$0.6, smaller than that reported for carbon nanotubes (1.40). This difference may be ascribed to the modified Coulomb potential in two dimensions. To further elucidate this power-law scaling of 1s excitons, experiments with encapsulating materials with dielectric constants different from hBN are needed, though, to the best of our knowledge, there is no material reported to offer sample quality as high as hBN at the present. Theoretically, it will be necessary to conduct time-dependent first-principles calculations of exciton energy level structure to investigate how it is affected by material characteristics, such as the band structure, electronic wave function, and Berry curvature^23,42–45^.

## Electronic Supplementary Material

Below is the link to the electronic supplementary material.

Supplementary Material 1

## Data Availability

Data underlying the results presented in this paper are available online (http://hdl.handle.net/2433/289127).
