# Author Correction: 3D hydrogen-like screening effect on excitons in hBN-encapsulated monolayer transition metal dichalcogenides

**DOI:** 10.1038/s41598-024-83150-8

**Published:** 2025-01-14

**Authors:** S. Takahashi, S. Kusaba, K. Watanabe, T. Taniguchi, K. Yanagi, K. Tanaka

**Affiliations:** 1 Department of Physics, Kyoto University, Kitashirakawa-Oiwake-cho, Sakyo-ku, Kyoto, 606-8502 Japan; 2 Research Center for Electronic and Optical Materials, National Institute for Materials Science, 1-1 Namiki, Tsukuba, 305-0044 Japan; 3 Research Center for Materials Nanoarchitectonics, National Institute for Materials Science, 1-1 Namiki, Tsukuba, 305-0044 Japan; 4 Department of Physics, Tokyo Metropolitan University, 1-1 Minami-Osawa, Hachioji, Tokyo 192-0397 Japan; 5 Institute for Integrated Cell-Material Sciences, Kyoto University, Yoshida-Ushinomiya-cho, Sakyo-ku, Kyoto, 606-8502 Japan

Correction to: *Scientific Reports* 10.1038/s41598-024-77625-x, published online 8 November 2024

The original version of this Article contained an error in References 27, 28, 30, 45 and 57, which were incorrectly given as:

“27. Voigt, J., Spiegelberg, F. & Senoner, M. Band parameters of CdS and CdSe single crystals determined from optical exciton spectra. Phys. Status Solidi 91, 189–199 (1979).

28. Senger, R. T. & Bajaj, K. K. Binding energies of excitons in II-VI compound-semiconductor based quantum well structures. Phys. Status Solidi Basic. Res. 241, 1896–1900 (2004).

30. Yang, Z. et al. Unraveling the exciton binding energy and the dielectric constant in single-crystal methylammonium lead tri-iodide perovskite. J. Phys. Chem. Lett. 8, 1851–1855 (2017).

45. Bieniek, M., Sadecka, K., Szulakowska, L. & Hawrylak, P. Theory of excitons in atomically thin semiconductors: Tight-binding approach. Nanomaterials 12, 1584 (2022).

57. Wilson, N. P., Finley, J. J. & Dery, H. Breakdown of the static dielectric screening approximation of Coulomb interactions in atomically thin semiconductors. Preprint at arXiv:2402.18639 (2024).”

The correct References are listed below:

“27. Voigt, J., Spiegelberg, F. & Senoner, M. Band parameters of CdS and CdSe single crystals determined from optical exciton spectra. Phys. Status Solidi (b) 91, 189–199 (1979).

28. Senger, R. T. & Bajaj, K. K. Binding energies of excitons in II-VI compound-semiconductor based quantum well structures. Phys. Status Solidi (b) 241, 1896–1900 (2004).

30. Yang, Z. et al. Unraveling the exciton binding energy and the dielectric constant in single-crystal methylammonium lead triiodide perovskite. J. Phys. Chem. Lett. 8, 1851–1855 (2017).

45. Bieniek, M., Sadecka, K., Szulakowska, L. & Hawrylak, P. Theory of excitons in atomically thin semiconductors: Tight-binding approach. Nanomaterials 12, 1582 (2022).

57. Mhenni, A. B. et al. Breakdown of the static dielectric screening approximation of Coulomb interactions in atomically thin semiconductors. Preprint at arXiv:2402.18639 (2024).”

In addition, the legend of Table 1 contained an error in the spectral resolution.

As a result, the legend of Table 1

“The errors of the experimental values are determined by the spectral resolution (∼6 meV) and the fitting error. The experimental data for 1L-WSe_2_ are from our previous report^20^.”

now reads,

“The errors of the experimental values are determined by the spectral resolution (∼0.6 meV) and the fitting error. The experimental data for 1L-WSe_2_ are from our previous report^20^.”

The original Article has been corrected.

