# Supplementary material for: 3D hydrogen-like screening effect on excitons in hBN-encapsulated monolayer transition metal dichalcogenides
Source: Sci Rep. 2024 Nov 8;14:27286. doi: 10.1038/s41598-024-77625-x (PMC11549476; doi:10.1038/s41598-024-77625-x)
Supplement: Supplementary file 1 — Supplementary Material 1 [file 41598_2024_77625_MOESM1_ESM.docx]

**Supplementary information**

S. Takahashi^1,*^, S. Kusaba^1^, K. Watanabe^2^, T. Taniguchi^3^,

K. Yanagi^4^, and K. Tanaka^1,5,*^

^1^ *Department of Physics, Kyoto University, Kyoto, 606-8502, Japan.*

*^2^ Research Center for Electronic and Optical Materials, National Institute for Materials Science, 1-1 Namiki, Tsukuba 305-0044, Japan.*

*^3^ Research Center for Materials Nanoarchitectonics, National Institute for Materials Science, 1-1 Namiki, Tsukuba 305-0044, Japan.*

*^4^ Department of Physics, Tokyo Metropolitan University, Hachioji, Tokyo, 192-0397, Japan.*

*^5^ Institute for Integrated Cell-Material Sciences, Kyoto University, Kyoto, 606-8502, Japan.*

*e-mail: takahashi.shinya.87v@st.kyoto-u.ac.jp, kochan@scphys.kyoto-u.ac.jp

# S1. Sample preparation and characterization

hBN-encapsulated 1L-TMD samples are prepared by mechanical exfoliation and dry transfer on 300-nm SiO_2_/Si substrates. MoS_2_ and WS_2_ samples are prepared in Ar gas, and the other samples prepared in ambient conditions. After each transfer, annealing in 4% H_2_:Ar gas atmosphere is conducted for 2 hours. The optical images of MoS_2_, MoSe_2_, and WS_2_ samples are displayed in Figs. S1(a-c). The thicknesses of bottom hBN are estimated by reflection measurements to be around 342, 350, and 220 nm for MoS_2_, MoSe_2_, and WS_2_ samples, respectively. The thicknesses of top hBN are around 8 nm for the three samples. Photoluminescence intensity maps are shown in Figs. S1(d-f) for the indicated regions in Figs. S1(a-c) under excitation at 2.33 eV in ambient conditions. Figures S1(g-j) display Raman spectra in ambient conditions for four samples. The excitation wavelength is 532 nm. The peaks at $\sim$520 cm^-1^ are originated from Si. The peaks at $\sim$1365 cm^-1^ are E_2g_ mode of hBN. This peak position is considered reasonable though it is slightly deviated from the value reported in a previous report^S1^ possibly due to the difference of substrate. Broad tail observed for WS_2_ in Fig. S1(i) is considered that of exciton emission. In the following, we compare the Raman modes for 1L-TMDs with those in a previous report^S2^. The peak positions are slightly deviated from the value in the reference probably because they are shifted for a different substrate. For MoS_2_ in Fig. S1(g), two lines are observed at 384 cm^−1^ (E’ mode) and at 405 cm^−1^ (A1′). The difference between the two modes is 21 cm^−1^, which confirms that the sample is 1L-MoS_2_. The E′ line is found to have a full width at half-maximum (FWHM) value of 3.2 cm^−1^, which ensures a good crystallinity of MoS_2_. For MoSe_2_ in Fig. S1(h), a peak at 240 cm^−1^ (A_1g_) and one at 286 cm^−1^ (E_2g_) as well as the combination of LA(M) and TA(M) modes at 251 cm^−1^ are observed, which indicates that the sample is 1L-MoSe_2_. For WS_2_ in Fig. S1(i), peaks at 175 cm^−1^ (LA(M)), 350 cm^−1^ (2LA(M)), 418 cm^−1^ (A_1_’) are observed. The high intensity of the 2LA(M) peak under 532-nm excitation is also consistent with the previous report^S2^. These indicate that the sample is comprised of 1L-WS_2_. For WSe_2_ in Fig. S1(j), peaks at 248 cm^−1^ (A_1g_) and at 260 cm^−1^ (2LA(M)) are observed, which suggests that the sample is composed of 1L-WSe_2_.

#
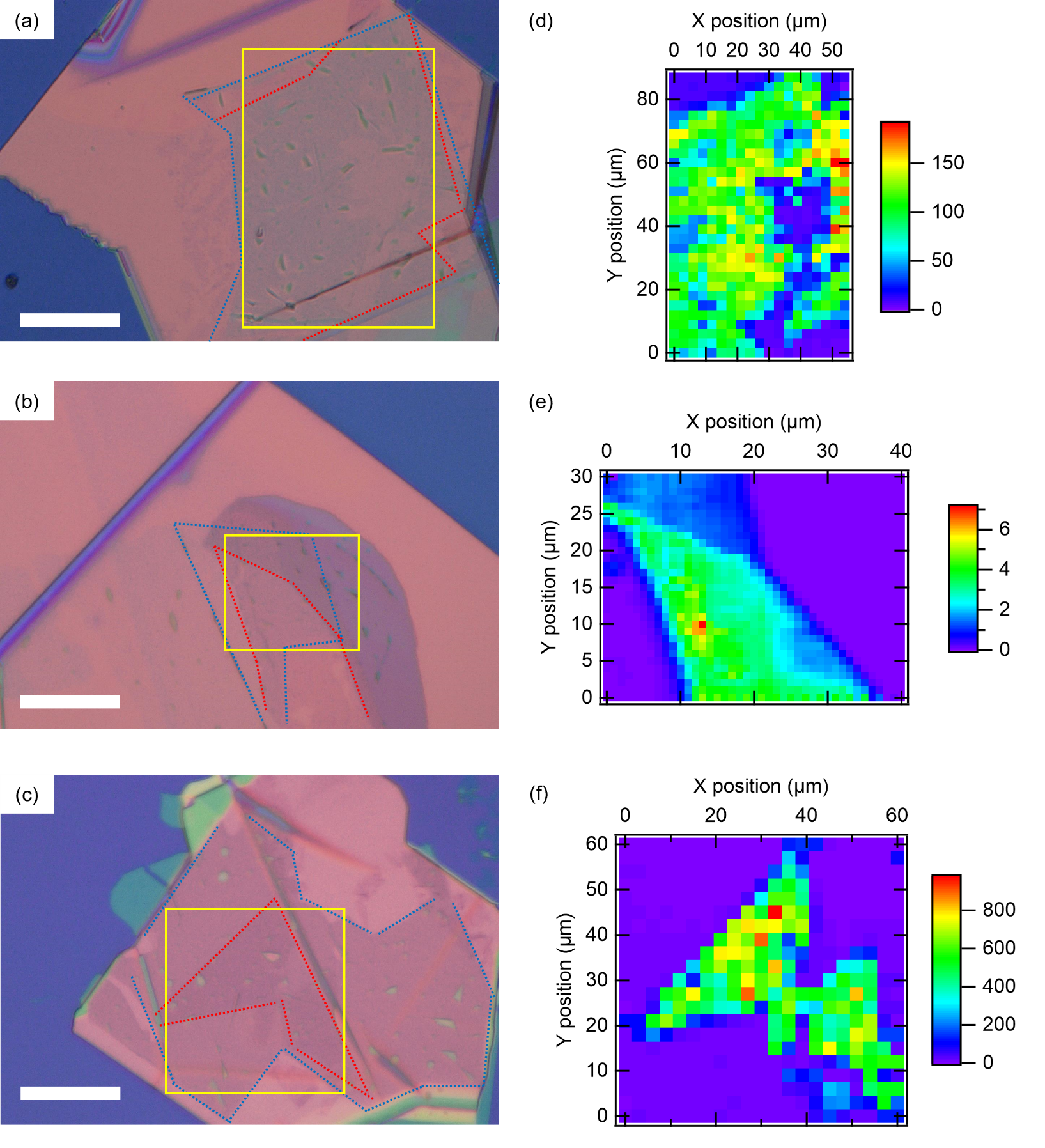


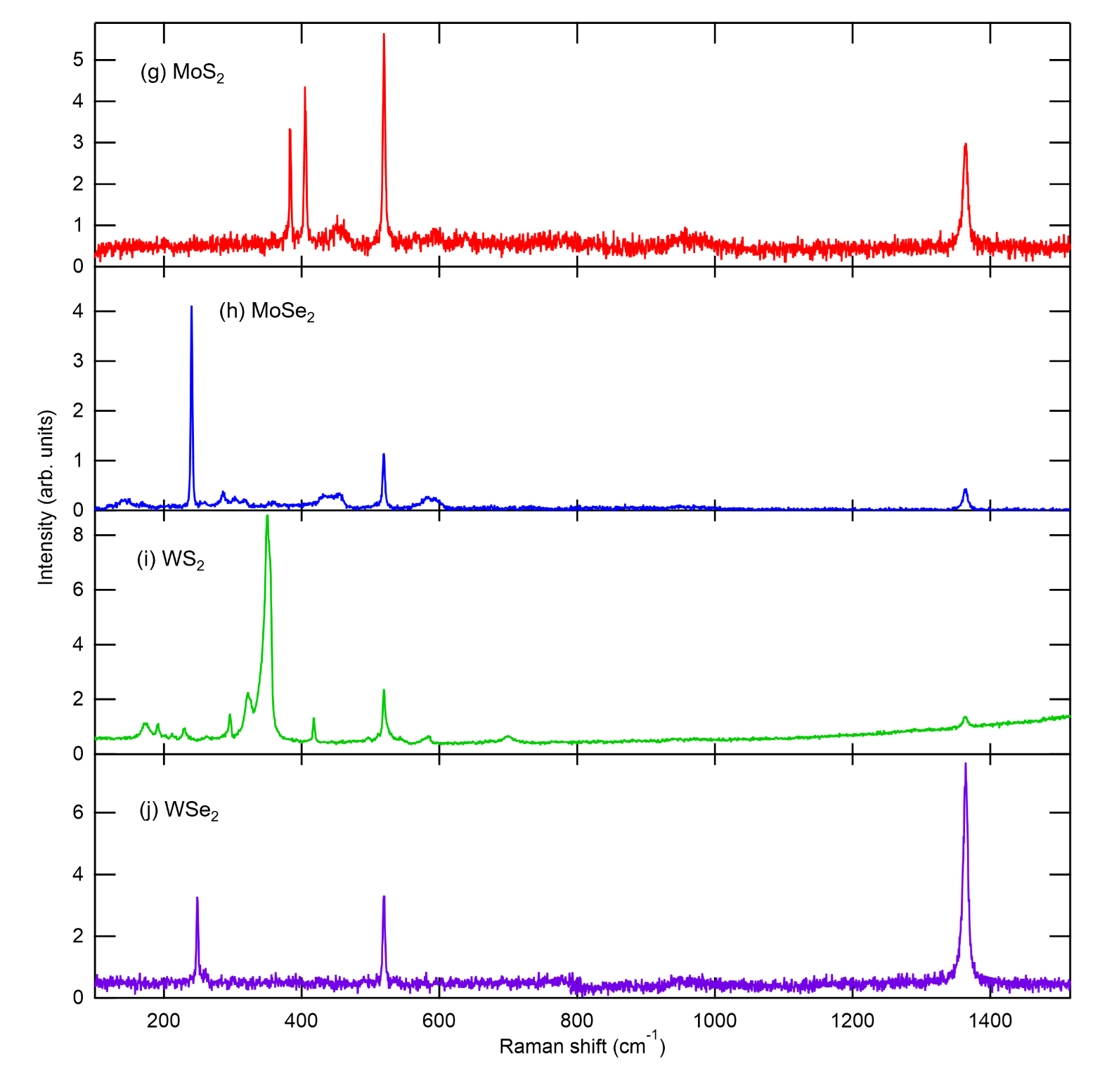


FIG. S1. Optical images of (a) 1L-MoS_2_, (B) 1L-MoSe_2_, and (C) 1L-WS_2_ samples. Red and blue lines correspond to 1L-TMDs and top hBN region, respectively. Scale bars are 30 μm (d-f) Photoluminescence intensity maps in the yellow squares in (a-c) under excitation at 2.33 eV in ambient conditions. Color bars are in arbitrary units. (g-j) Raman spectra for four hBN-encapsulated 1L-TMD samples. The excitation wavelength is 532 nm. Peaks around 200-400 cm^-1^ are from 1L-TMDs (detailed description in the text), those at $\sim$520 cm^-1^ from Si, and those at $\sim$1367 cm^-1^ from hBN, respectively. Broad tail observed for WS_2_ is considered that of exciton emission.

# S2. Peak assignments of nonlinear emission spectra and extraction of coherent components

The results of linear spectroscopies, and the power and polarization dependences of nonlinear emissions at low temperature are shown in Figs. S2, S3, and S4 for MoS_2_, MoSe_2_, and WS_2_ samples, respectively. In the linear spectroscopies, s-series excitons are observed, although p-series excitons are not observed. By comparing these results with the nonlinear emission spectra, each exciton level is assigned. The power dependences in the insets of Figs. S2(c), S3(c), and S4(c) are consistent with the ansatz that the observed peaks in Figs. S2-S4 are originated from nonlinear processes. There are, however, two contributions to the nonlinear emissions; coherent components originating from resonant sum frequency generation (SFG) and incoherent ones from two-photon photoluminescence (2P-PL), which involves relaxation after excitation. These two components can be differentiated by measuring the emission intensities parallel and perpendicular to the excitation polarization under excitation along the armchair and zigzag directions. This is because coherent components of resonant SFG follow the selection rule of second-order nonlinearity of 1L-TMDs while incoherent ones of 2P-PL do not have polarization dependence or simply have polarization parallel to the excitation. As shown in our previous report^S3^, the proportion of resonant SFG can be estimated by the averaged value:

$$\frac{DOP^{\left( \mathrm{Arm}. \right)}-DOP^{\left( Zig. \right)}}{2},$$

where degree of polarization $DOP$ under excitation along armchair / zigzag directions is calculated by $({I_{\parallel}-I}_{\perp})/({I_{\parallel}+I}_{\perp})$ by using emission intensities $I_{\parallel}$ ($I_{\perp}$) parallel (perpendicular) to the excitation polarization. The residual proportions are attributed to 2P-PL. From the nonlinear emission data in Figs. S2(d), S2(e), S3(d), S3(e), S4(d), and S4(e), we differentiate two processes in each material as tabulated in Table S1. For the other peaks, trions are found to be dominated by 2P-PL in MoS_2_, MoSe_2_, and WS_2_. B:1s excitons, which are derived from another band-to-band transition, are also observed in MoSe_2_ and weakly in MoS_2_, and found to be originated mostly from SFG processes in both materials.


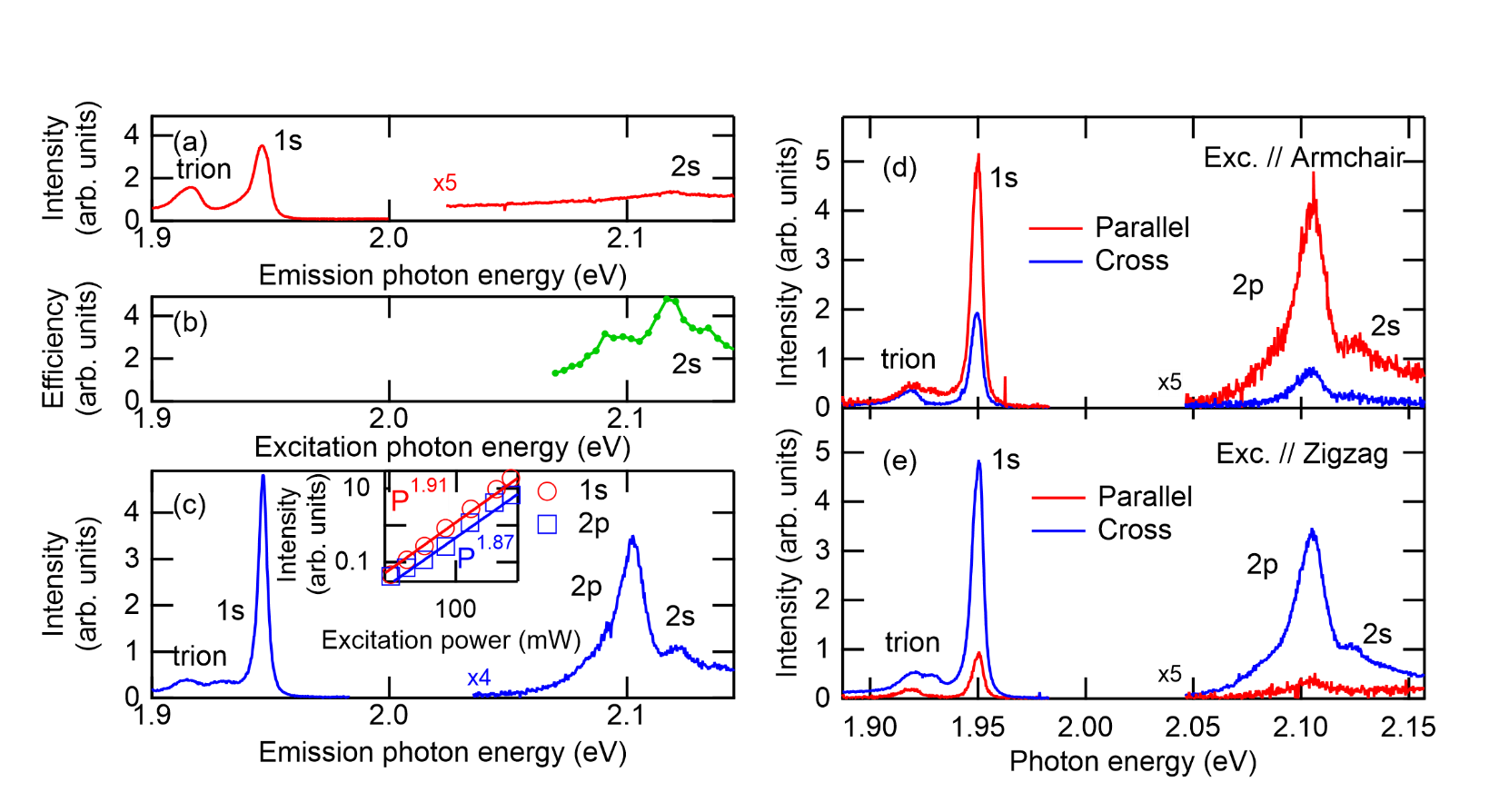


FIG. S2. Results of spectroscopies for 1L-MoS_2_ at 10 K. (a) Photoluminescence spectrum under excitation at 2.34 eV. (b) Photoluminescence excitation spectrum observed at 1s exciton peak (1.95 eV). (c) Nonlinear emission spectrum, which is the same as in Fig. 2(c). The inset shows the dependences excitation power. Nonlinear emission spectra under excitation of linear polarizations along (d) the armchair and (e) the zigzag directions of the sample. The detection polarizations are set parallel and cross to the excitation polarizations.


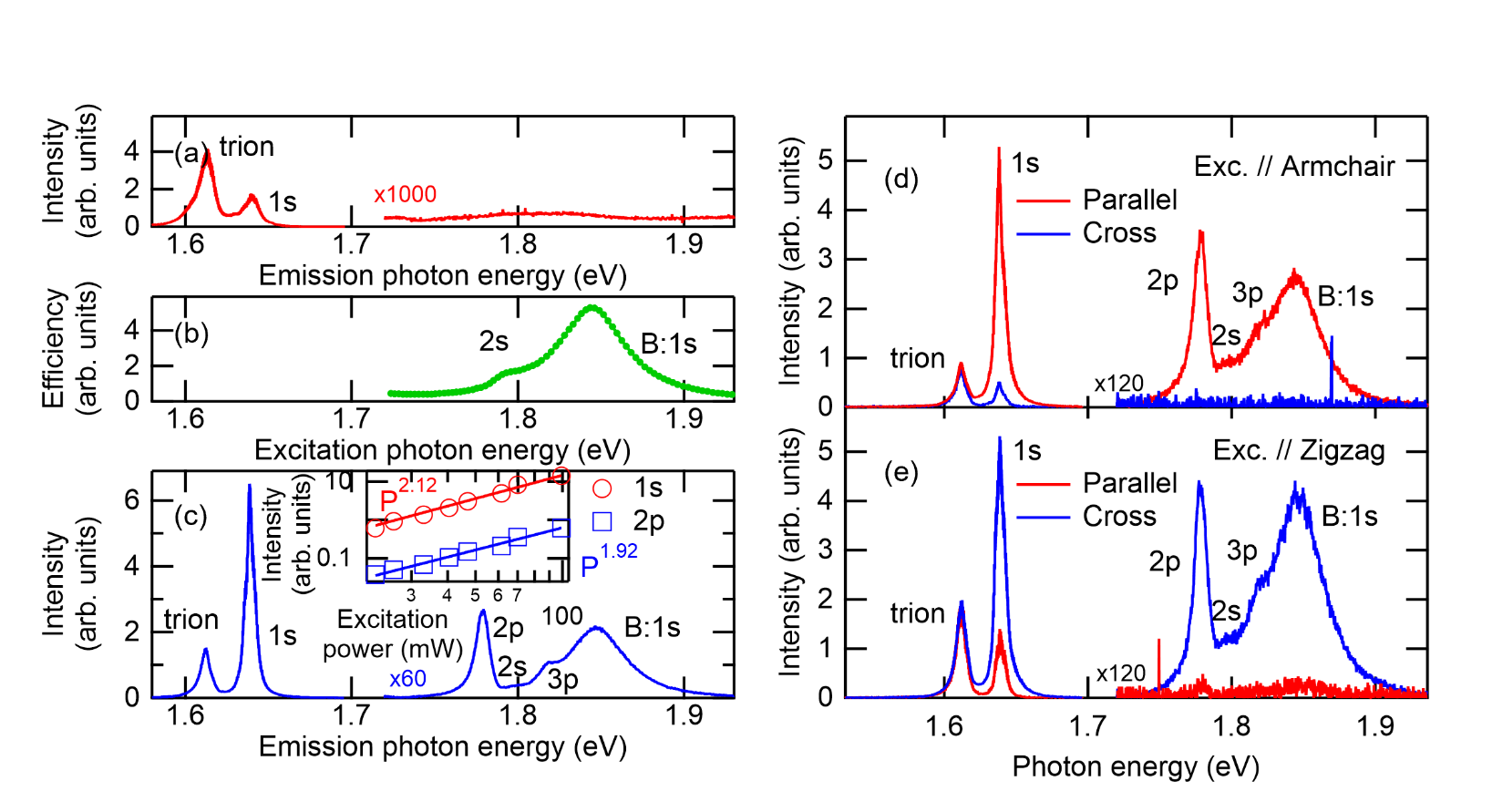


FIG. S3. Results of spectroscopies for 1L-MoSe_2_ at 10 K. (a) Photoluminescence spectrum under excitation at 2.34 eV. (b) Photoluminescence excitation spectrum observed at trion peak (1.61 eV). (c) Nonlinear emission spectrum, which is the same as in Fig. 2(a). The inset shows the dependences excitation power. Nonlinear emission spectra under excitation of linear polarizations along (d) the armchair and (e) the zigzag directions of the sample. The detection polarizations are set parallel and cross to the excitation polarizations.


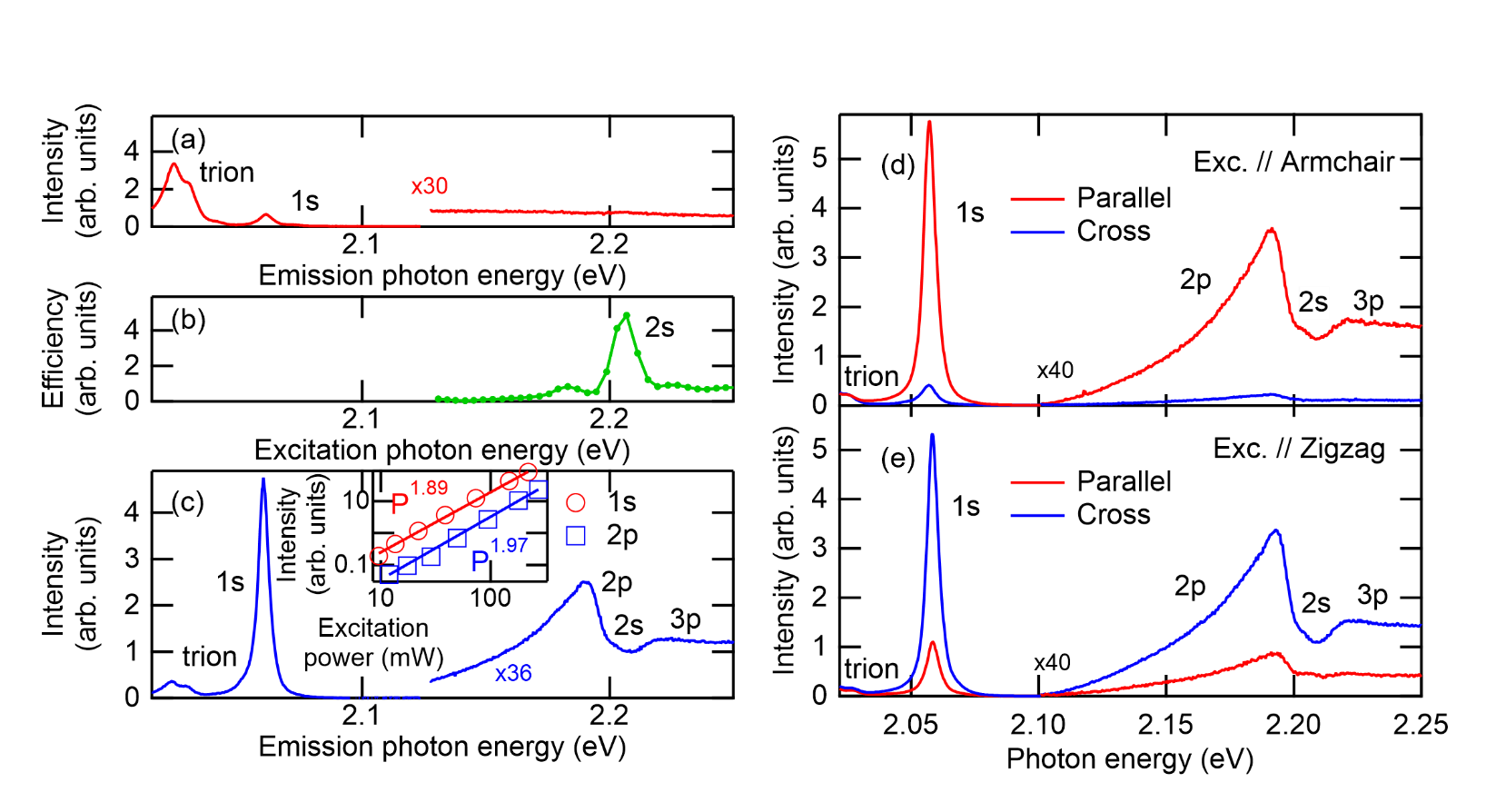


FIG. S4. Results of spectroscopies for 1L-WS_2_ at 10 K. (a) Photoluminescence spectrum under excitation at 2.53 eV. (b) Photoluminescence excitation spectrum observed at 1s exciton peak (2.06 eV). (c) Nonlinear emission spectrum, which is the same as in Fig. 2(d). The inset shows the dependences excitation power. Nonlinear emission spectra under excitation of linear polarizations along (d) the armchair and (e) the zigzag directions of the sample. The detection polarizations are set parallel and cross to the excitation polarizations.

| Orbital | Proportion of SFG components (%) | | | |
| --- | --- | --- | --- | --- |
|  | MoS_2_ | MoSe_2_ | WS_2_ | WSe_2_ |
| 1s | 57.0$\pm$0.4 | 74.4$\pm$0.2 | 72.9$\pm$0.1 | 56.8$\pm$0.4 |
| 2p | 70.0$\pm$2.0 | 94.1$\pm$0.9 | 76.9$\pm$0.9 | 96.7$\pm$1.1 |
| 2s | 73.6$\pm$15.9 | 90.4$\pm$9.8 | 26.6$\pm7$.8 | 18.8$\pm$2.0 |
| 3p |  | 92.0$\pm$3.0 | 69.0$\pm$4.1 | 66.3$\pm$10.3 |
| 3s |  |  |  | 47.0$\pm$7.2 |
| B:1s | 71.8$\pm$2.4 | 92.3$\pm$1.5 |  |  |

Table S1. Proportion of coherent SFG components deduced from the polarization dependences. The data of WSe_2_ are from our previous report^S3^.

# S3. Estimation of unintentionally doped carriers in the samples

As described in section S2, we observe intense emissions of trions in the PL spectra especially for MoSe_2_ and WS_2_. To confirm that unintentionally doped carriers do not influence the exciton level structure, we estimate the densities of carriers in our samples as described in the next paragraph. First, it should be noted that the physical origin of the emission lines conventionally called “trion” is still disputed. In previous report^S4^, it was reported that exciton polaron appears by carrier doping, and that exciton polaron and excitons show a significant energy shift for carrier doping above 10^11^ cm^-2^. Their structure is also reported to appear in the reflection spectrum reflecting absorption. Recently, an increasing number of authors have considered exciton polaron instead of “trion” in samples with high-density carrier doping, which is consistent with the dielectric screening effect of carriers. However, to avoid confusion, we will refer to the spectral component appearing on the lower-energy side of the exciton as trion in this paper.

To estimate the carrier densities of our samples, we refer the peak ratio between 1s exciton and trion in the reflectance^S4^. First, we consider the squares of the transition matrix elements for reflection and resonant SFG processes for 1s states, by using dipole momentum operator $\hat{d}$ as^S3^

$$\begin{aligned} M_{1}=\left| \left\langle1s\left| \hat{d} \right|g \right\rangle\right|^{2}, \#\left( S1 \right) \end{aligned}$$

and

$$\begin{aligned} \left| \sum_{i=2p, 3p,\cdots} \left\langle g\left| \hat{d} \right|1s \right\rangle\left\langle1s\left| \hat{d} \right|i \right\rangle\left\langle i\left| \hat{d} \right|g \right\rangle\right|^{2}=M_{1}\left| \sum_{i=2p, 3p,\cdots} \left\langle1s\left| \hat{d} \right|i \right\rangle\left\langle i\left| \hat{d} \right|g \right\rangle\right|^{2}, \#\left( S2 \right) \end{aligned}$$

respectively, where $|g\rangle$ is the ground state. This indicates that the square of the transition matrix element for the resonant SFG process is proportional to that of the reflection (or absorption) and the internal transition term. Next, we assume that these equations (S1) and (S2) hold for both excitons and trions and the internal transition term be the same. Only difference should be the carrier density contribution that is an overall factor multiplied to eqs. (S1) and (S2). We make additional assumption that this overall factor is also the same for both excitons and trions. Under this assumption, the peak ratios of resonant SFG and reflection should be the same and reflect the carrier density. From this point of view, we can roughly estimate the carrier densities of our samples by comparing the coherent component ratio of the 1s exciton and trion in the nonlinear emission spectra in Figs. S3(c) and 4(c) with the peak ratio of reflection reported in ref. S4.

According to the results for MoSe_2_ in Figs. 3(g) and 4(c) of ref. S4, in the large carrier density condition, e.g. exciton energy shift occurs, where the dielectric screening of carriers matters. The threshold for the carrier screening effect is ~10^11^ cm^-2^ and the peak ratio of exciton / trion should be around 5 for this carrier density. For lower densities, the peak ratio of exciton / trion is expected to increase linearly.

As for SFG, we take MoSe_2_ as an example. By the spectral fitting with two Lorentzian functions to the nonlinear emission spectrum in Fig. S3(c), the intensity ratio of exciton / trion is found to be 3.9. However, we cannot use this value for our purpose since the ratio includes contributions of both resonant SFG and 2P-PL as described in section S2. We make fittings to the polarization-resolved spectra in Figs. S3(d-e) to obtain the ratio of the resonant SFG component. As for 1s exciton levels, we obtain 74.4$\pm$0.2% as shown in Table S1. Similarly to the calculation processes described in section S2, we determine the proportion of coherent component of trion as 0.0$\pm$0.1%. Therefore, we obtain the coherent component ratio of exciton / trion as ~3$\times$10^3^ at least. The estimation for WS_2_ can similarly be obtained as ~4$\times$10^3^ at least. By comparing these values with the peak ratio in reflection^S4^, we consider that the carrier densities of our samples are at least two orders of magnitude smaller than the threshold, that is, <~10^9^ cm^-2^. This estimation confirms negligible carrier effect.

We observe intense trion emissions in the PL spectra in Figs. S3(a) and S4(a), but because the peak ratio of the 1s exciton and trion can vary widely depending on the sample temperature and excitation condition, it is difficult to make fair estimation (e.g. refer to previous studies^S5, S6^ for W-based 1L-TMDs). Actually, the excitation conditions in our PL measurements are above band gap and a number of carriers are photo-doped, which may lead to sizable trion PL emissions.

# S4. Fitting functions in nonlinear emission spectra

As described in eq. (1) in the main text, the fitting functions in Figs. 2(a-d) have the following form:

$$S(\omega)=(coherent part)+(incoherent part)$$

$$\begin{aligned} =\left| a+b\hbar\omega+\sum_{j} \frac{\sqrt{A_{j}}}{\hbar\omega-\epsilon_{j}+i\frac{\gamma_{j}}{2}} \right|^{2}+\sum_{j} \frac{B_{j}}{\left( \hbar\omega-\epsilon_{j} \right)^{2}+\left( \frac{\gamma_{j}}{2} \right)^{2}}.\#\left( S3 \right) \end{aligned}$$

The coherent part corresponds to resonant SFG. The linear part in the coherent part is non-resonant component. The incoherent part is originated from 2P-PL. 1s exciton peaks are found to be dominated by SFG processes in all 1L-TMDs, but they are separated by the other peaks. That is why we use usual Lorentz function for the fitting functions to the 1s exciton peaks. The fitting functions are expressed as follows:

For all 1L-TMDs around the 1s exciton,

$$\frac{B_{1s}}{\left( \hbar\omega-\epsilon_{1s} \right)^{2}+\left( \gamma_{1s}/2 \right)^{2}}.$$

For MoS_2_ around the 2p and 2s excitons,

$$\left| a+b\hbar\omega+\sum_{j=2p,2s,B:1s} \frac{\sqrt{A_{j}}}{\hbar\omega-\epsilon_{j}+i\gamma_{j}/2} \right|^{2}.$$

For MoSe_2_ around the 2p and 2s excitons,

$$\left| a+b\hbar\omega+\sum_{j=2p,2s,3p,B:1s} \frac{\sqrt{A_{j}}}{\hbar\omega-\epsilon_{j}+i\gamma_{j}/2} \right|^{2}.$$

For WS_2_ around the 2p and 2s excitons,

$$\left| a+b\hbar\omega+\sum_{j=2p,3p} \frac{\sqrt{A_{j}}}{\hbar\omega-\epsilon_{j}+i\gamma_{j}/2} \right|^{2}+\frac{B_{2s}}{\left( \hbar\omega-\epsilon_{2s} \right)^{2}+\left( \gamma_{2s}/2 \right)^{2}}.$$

For WSe_2_ around the 2p and 2s excitons,

$$\left| a+b\hbar\omega+\sum_{j=2p,3p} \frac{\sqrt{A_{j}}}{\hbar\omega-\epsilon_{j}+i\gamma_{j}2} \right|^{2}+\sum_{j=2s,3s} \frac{B_{j}}{\left( \hbar\omega-\epsilon_{j} \right)^{2}+\left( \gamma_{j}/2 \right)^{2}}.$$

The resultant parameters of the fittings are summarized in Table S2-S5, and the example of the fitting for 1L-MoSe_2_ is displayed in Fig. S5.


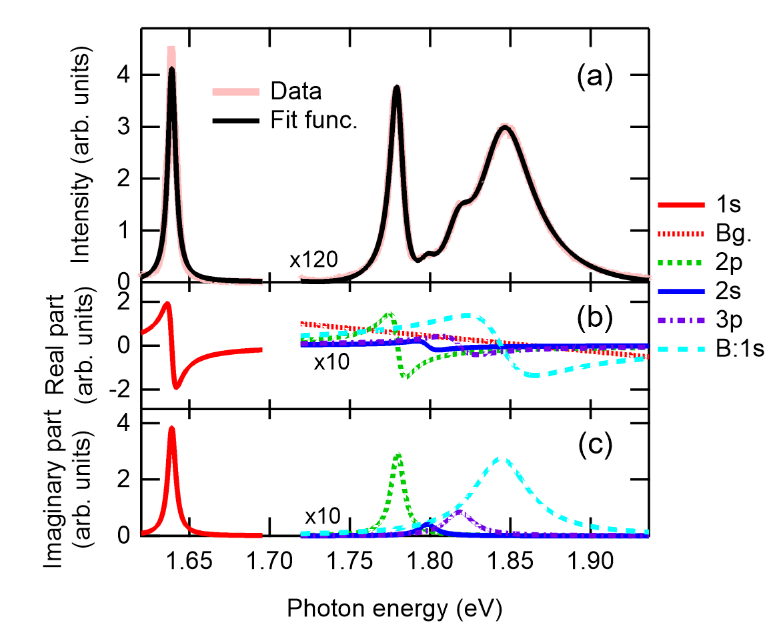


FIG. S5. Fitting components for 1L-MoSe_2_ in Fig. 2(a). (a) The data and total fitting function, (b) real parts, and (c) imaginary parts are shown.

| Orbital | Intensity $A$ / $B$ (eV^2^) | Energy $\epsilon$ (eV) | FWHM $\gamma$ (meV) |
| --- | --- | --- | --- |
| 1s | 0.00212$\pm$0.00009 | 1.9466$\pm$0.0006 | 4.2$\pm0.6$ |
| 2p | 0.00102$\pm$0.00005 | 2.1038$\pm$0.0006 | 11.8$\pm$0.6 |
| 2s | 0.000002$\pm$0.000003 | 2.1229$\pm$0.0007 | 7$\pm$1 |
| B:1s | 0.032$\pm$0.002 | 2.125$\pm$0.0015 | 95$\pm$4 |

Table S2. Parameters of fitting functions in 1L-MoS_2_. FWHM is full width at half maximum. $a=128\pm4,b=-62\pm2$ eV^-1^.

| Orbital | Intensity $A$ / $B$ (eV^2^) | Energy $\epsilon$ (eV) | FWHM $\gamma$ (meV) |
| --- | --- | --- | --- |
| 1s | 0.051$\pm$0.001 | 1.6390$\pm$0.0006 | 5.9$\pm$0.6 |
| 2p | 0.0098$\pm$0.0002 | 1.7797$\pm$0.0006 | 9.8$\pm$0.6 |
| 2s | 0.00035$\pm$0.0003 | 1.7983$\pm$0.0006 | 13$\pm$1 |
| 3p | 0.0037$\pm$0.003 | 1.8182$\pm$0.0007 | 20.8$\pm$0.9 |
| B:1s | 0.158$\pm$0.003 | 1.844$\pm$0.001 | 42$\pm$0.7 |

Table S3. Parameters of fitting functions in 1L-MoSe_2_. $a=90\pm1,b=-48.3\pm0.7$ eV^-1^.

| Orbital | Intensity $A$ / $B$ (eV^2^) | Energy $\epsilon$ (eV) | FWHM $\gamma$ (meV) |
| --- | --- | --- | --- |
| 1s | 0.0099$\pm$0.0004 | 2.0601$\pm$0.0006 | 5.0$\pm$0.6 |
| 2p | 0.0092$\pm$0.0002 | 2.1944$\pm$0.0006 | 18.6$\pm$0.6 |
| 2s | 0.00008$\pm$0.00003 | 2.2033$\pm$0.0006 | 4$\pm$1 |
| 3p | 0.0058$\pm$0.0007 | 2.2272$\pm$0.0007 | 38$\pm$2 |

Table S4. Parameters of fitting functions in 1L-WS_2_. $a=215\pm2,b=-104.1\pm0.7$ eV^-1^.

| Orbital | Intensity $A$ / $B$ (eV^2^) | Energy $\epsilon$ (eV) | FWHM $\gamma$ (meV) |
| --- | --- | --- | --- |
| 1s | 0.0098$\pm$0.0004 | 1.6982$\pm$0.0006 | 3.8$\pm$0.6 |
| 2p | 0.00206$\pm$0.00006 | 1.8203$\pm$0.0006 | 8.5$\pm$0.6 |
| 2s | 0.00107$\pm$0.00003 | 1.8273$\pm$0.0006 | 5.0$\pm$0.6 |
| 3p | 0.00025$\pm$0.00007 | 1.8453$\pm$0.0007 | 8.8$\pm$0.8 |
| 3s | 0.0014$\pm$0.0002 | 1.8489$\pm$0.0008 | 10.8$\pm$0.8 |

Table S5. Parameters of fitting functions in 1L-WSe_2_. $a=-137\pm7,b=78\pm4$ eV^-1^.

# S5. Evaluation of parameters in numerical calculations

The values of the relative dielectric constant of hBN $\kappa$, the screening length of 1L-TMD $r_{0}$, and the binding energy of the 1s exciton $E_{b}^{1s}$ are evaluated by the deviation of exciton energies in the numerical calculation from those in the experiment. The detailed procedure is (i) evaluation of the central values of the parameters $\kappa$, $r_{0}$, and $E_{b}^{1s}$, (ii) fitting to the deviation profile of exciton energies, (iii) evaluation of total errors of exciton energies, and (iv) evaluation of the errors of $\kappa$ and $r_{0}$.

Firstly, the central values of $\kappa$ and $r_{0}$ are evaluated by calculating the deviation of exciton energies in the numerical calculation from those in the experiment. Deviation of exciton energies is calculated by

$$\begin{aligned} \sqrt{\frac{1}{n_{\mathrm{stat}}-2}\sum_{j=2p,2s,\ldots} \left( \Delta\epsilon_{\mathrm{calc}}^{j}-\Delta\epsilon_{\exp}^{j} \right)^{2}}, \#\left( S4 \right) \end{aligned}$$

where $n_{\mathrm{stat}}$ is the number of states in the sum of $j=2p,2s,\ldots$, $-2$ in the denominator represents the number of parameters ($\kappa$, $r_{0}$) to adjust calculated exciton energies to the experimental values, and $\Delta\epsilon^{j}$ is the energy difference between the 1s and another level $j=2p,2s,\ldots$ in the calculation or the experiment. This is a normalized form of eq. (3) in the text to express the deviation for one exciton level. Figures S6(a,b) show representative results of evaluation of the deviation in eq. (S4) for 1L-WSe_2_ for different scales of $\kappa$ and $r_{0}$. The minimal position in the map is set to the central values of $\kappa$ and $r_{0}$ and the minimal value is taken as the error in the numerical calculation $\sigma_{\mathrm{calc}}$. We do not take account of the error of exciton reduced mass. For MoS_2_, it is impossible to evaluate $\sigma_{\mathrm{calc}}$ and eq. (S4) because $n_{\mathrm{stat}}=2$. Hence, we change the fraction in eq. (S4) to $1/2$ and do not take account of $\sigma_{\mathrm{calc}}$.

Secondly, fitting to the deviation maps as shown in Figs. S6(a,b) is performed. From the map profiles, it is better to transform the coordinates $(\kappa, r_{0})$ to

$$\xi=\frac{\alpha\kappa+r_{0}}{\sqrt{1+\alpha^{2}}},$$

and

$$\eta=\frac{\kappa-\alpha r_{0}}{\sqrt{1+\alpha^{2}}},$$

where $r_{0}$ is made dimensionless with unit of 1 nm. The fitting function in this coordinate is

$$\begin{aligned} f\left( \xi,\eta\right)=f_{0}+C_{\xi1}\left( \xi-\xi_{0} \right)+C_{\xi2}\left( \xi-\xi_{0} \right)^{2}+C_{\eta1}\left( \eta-\eta_{0} \right)+C_{\eta2}\left( \eta-\eta_{0} \right)^{2},\#\left( S5 \right) \end{aligned}$$

where $\xi_{0}$ and $\eta_{0}$ correspond to the central values of $\kappa$ and $r_{0}$. We let different coefficients for different directions (e.g. for $\xi>0$ and $\xi<0$). The results of the fitting to Figs. S6(a,b) are shown in Figs. S6(c,d), respectively, and the fitting functions are found to reproduce the profiles well.

Thirdly, total errors of exciton energies $\sigma_{\mathrm{tot}}$ are evaluated by using the error in numerical calculation $\sigma_{\mathrm{calc}}$ and that in experiment $\sigma_{\exp}$:

$$\sigma_{\mathrm{tot}}=\sqrt{\sigma_{\mathrm{calc}}^{2}+\sigma_{\exp}^{2}}.$$

$\sigma_{\exp}$ is evaluated by

$$\sigma_{\exp}=\sqrt{\frac{1}{n_{\mathrm{stat}}+1}\sum_{j=1s, 2p,2s,\ldots} {\sigma_{\exp}^{j}}^{2}},$$

where $n_{\mathrm{stat}}$ is the same as in eq. (S4), $+1$ comes from the contribution of 1s, and $\sigma_{\exp}^{j}$ is the error of energy for each exciton level $j$, which is shown in the upper panel of Table 1 in the text. The result of $\sigma_{\mathrm{tot}}$ is taken as the error of the 1s binding energy $E_{b}^{1s}$ and the band-gap energy $E_{g}$. For MoS_2_, we only consider $\sigma_{\exp}$.

Finally, the errors of $\kappa$ and $r_{0}$ are evaluated by using the fitting results and $\sigma_{\mathrm{tot}}$. We calculate the error of $\xi$ and $\eta$ by using the coefficients in eq. (S5). For example, for $\xi>0$, the error $\Delta\xi_{+}$ is calculated by

$$C_{\xi1+}\Delta\xi_{+}+C_{\xi2+}\left( \Delta\xi_{+} \right)^{2}=\sigma_{\mathrm{tot}}.$$

The error $\Delta\xi_{-}$ for $\xi<0$ is calculated similarly, and the error of $\xi$ is calculated by

$$\Delta\xi=\sqrt{\frac{\left( \Delta\xi_{+} \right)^{2}+\left( \Delta\xi_{-} \right)^{2}}{2}}.$$

The error of $\eta$ is calculated similarly. Eventually, the errors of $\kappa$ and $r_{0}$ are calculated by

$$\Delta\kappa=\sqrt{\frac{\left( \alpha\Delta\xi\right)^{2}+\left( \Delta\eta\right)^{2}}{1+\alpha^{2}}},$$

and

$$\Delta r_{0}=\sqrt{\frac{1}{1+\alpha^{2}}\left( \left( \Delta\xi\right)^{2}+\left( \alpha\Delta\eta\right)^{2} \right)}.$$

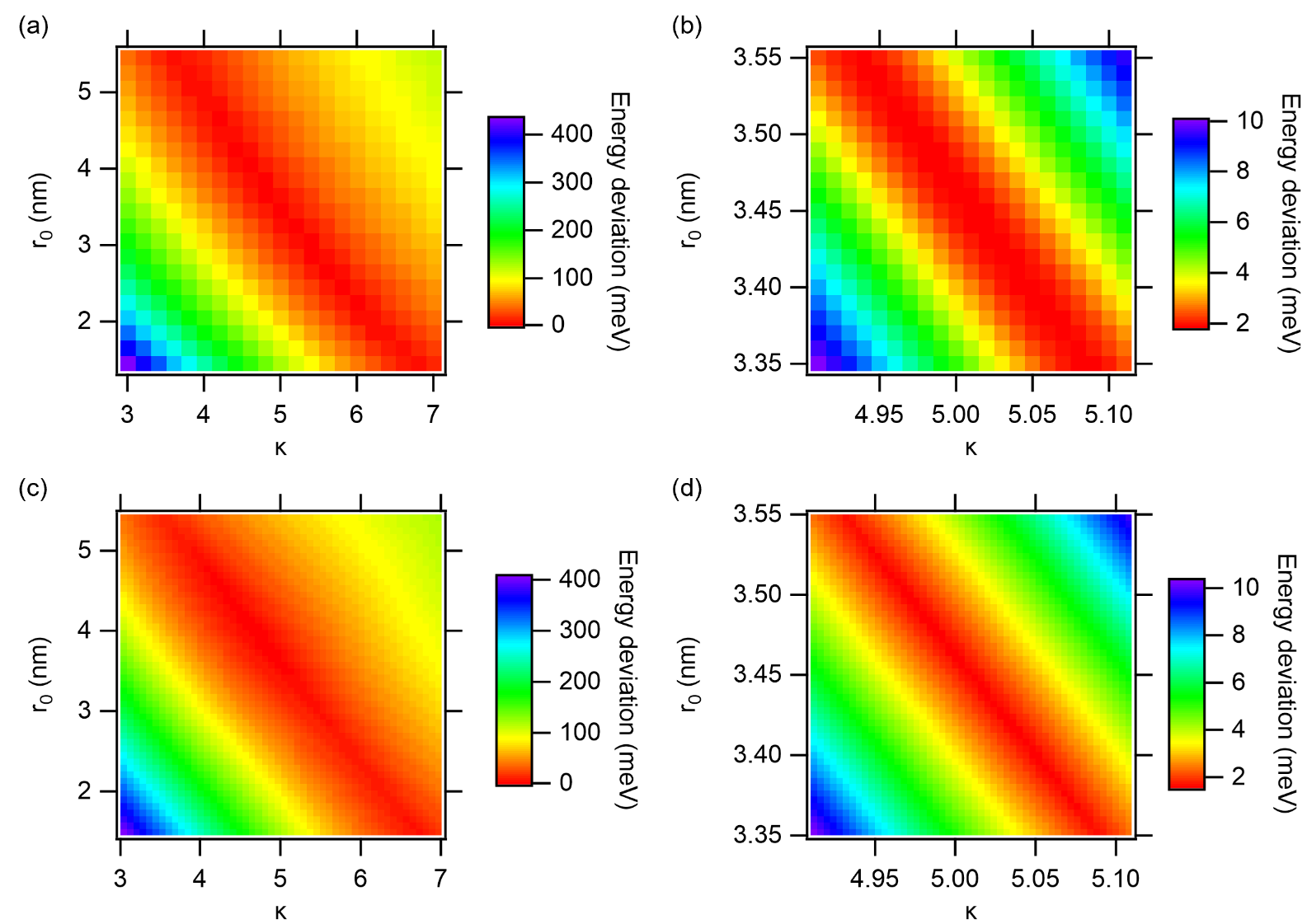


FIG. S6. Representative maps to evaluate the errors of the parameters for 1L-WSe_2_. (a, b) Deviation of exciton energies in the numerical calculation from those in the experiments (eq. (S4)) for different scales of $\kappa$ and $r_{0}$. (c, d) Results of the fitting (eq. (S5)) to (a, b) respectively.

# S6. Comparison of band-gap energy and binding energy with previous studies

Here, we compare the obtained band-gap energy and 1s exciton binding energy for hBN-encapsulated 1L-WSe_2_, whose binding energy has been reported most in the past, with those reported in previous studies^S7–S10^, as tabulated in Table S6. The values are found to disagree with each other with differences of ~20 meV, which is larger than the errors. The difference in measured temperatures is a concern, but the temperature dependence measurements in our previous paper^S3^ show that the temperature changes of band-gap energy and binding energy are smaller than the difference in Table S6. From this, we conclude that temperature is not the cause of this difference. The differences may originate from the slight difference in the dielectric environment of the sample^S11^, such as the distance between hBN and 1L-TMD. Similar differences from the reported values^S12^ are found for other 1L-TMDs.

It should be noted that these differences do not affect our conclusion including the power-law scaling in Fig. 4 of the main text as described in section S9.

|  | $\vert E_{b}^{1s}\vert$ (meV) | $E_{g}$ (eV) | $T$ (K) | Method |
| --- | --- | --- | --- | --- |
| This study | 164$\pm$1 | 1.862$\pm$0.001 | 16 | SFG |
| Stier *et al.* ^S7^ | 167 | 1.890 | 4 | Magneto-abs. |
| Liu *et al.* ^S8^ | 172 | 1.884 | 4 | Magneto-PL |
| Chen *et al.* ^S9^ | 170 | 1.901 | 2 | Magneto-PL |
| Wang *et al.* ^S10^ | 168.6 | 1.894 | 4.2 | Magneto-PC |

Table S6. Comparison of the obtained 1s exciton binding energy $|E_{b}^{1s}|$, band-gap energy $E_{g}$, sample temperature $T$, and measurement method for hBN-encapsulated 1L-WSe_2_ (abs. and PC are absorption and photocurrent, respectively).

# S7. Extended graph of Fig. 3(c)

In Fig. S7, the exciton binding energies are plotted against the relative dielectric constant of hBN including other levels than 1s. The solid line is energy dependence of the effective relative dielectric constant of hBN calculated by using expression in a theoretical paper^S13^ and parameter values in an experimental report^S14^, which is the same as in Fig. 3(c) of the main text.


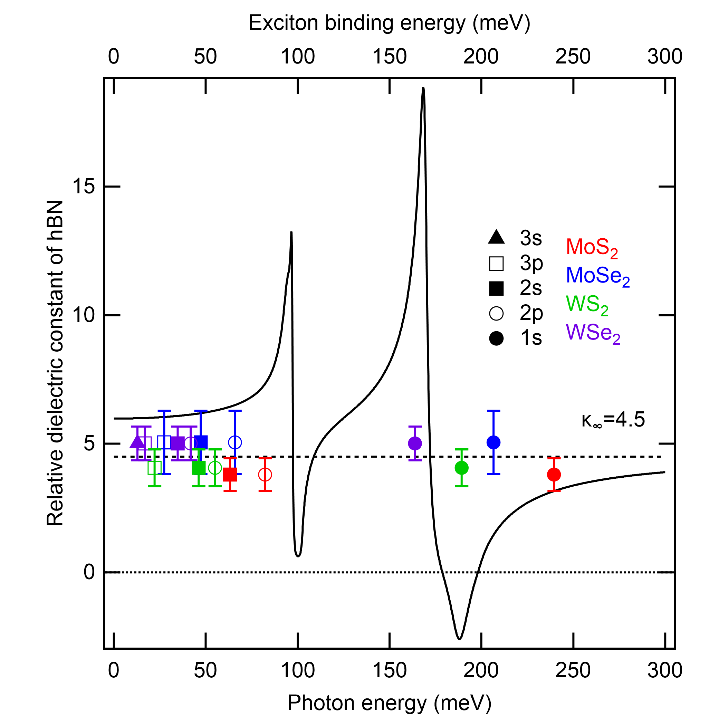


FIG. S7. Exciton binding energies (upper horizontal axis) against relative dielectric constant of hBN for four 1L-TMDs. The solid line is energy dependence (lower horizontal axis) of effective relative dielectric constant of hBN calculated by using expression in ref. S13 and parameter values in ref. S14. The errors of the plots are the same for the 1s exciton levels in the same 1L-TMD.

# S8. Power-law scaling for hydrogen model

For the 3D hydrogen model, binding energy $E_{b}$, Coulomb potential energy $E_{c}$, and kinetic energy $E_{k}$ can be expressed analytically. Firstly $E_{b}$ is expressed as

$$\begin{aligned} E_{b}=-\frac{Ry}{n^{2}}, \#\left( S6 \right) \end{aligned}$$

where $Ry=me^{4}/32\pi^{2}\varepsilon^{2}\hbar^{2}$ is the Rydberg constant, and $n$ is principal quantum number. This leads to $mR^{2}\left| E_{b} \right|\propto\left( mR/\varepsilon\right)^{2}$ for the 1s level, which was compared to $mR^{2}\left| E_{b} \right|\propto\left( mR/\varepsilon\right)^{\alpha}$ with $\alpha=1.40$ for carbon nanotube in the previous study^15^, where $m$, $R$, and $\varepsilon$ are exciton reduced mass, radius, and dielectric constant of the surroundings, respectively. Even if one compares $mR^{2}\left| E_{b} \right|$ and $mR/\varepsilon$ for the 3D hydrogen model, however, one cannot extract the relation; since only Bohr radius $a_{B}=4\pi\epsilon\hbar^{2}/me^{2}$ is a parameter with the dimension of length in the 3D hydrogen model, $mR^{2}\left| E_{b} \right|$ and $mR/\varepsilon$ are invariant for different $m$ or $\varepsilon$.

In this study, we rewrite the aforementioned relation as a power-law scaling $\left| E_{b} \right|/E_{k}\propto\left( {|E}_{c}|/E_{k} \right)^{\alpha}$, where $E_{c}$ is Coulomb potential energy ($=-e^{2}/4\pi\varepsilon R$), and $E_{k}$ is kinetic energy ($=\hbar^{2}/2mR^{2}$). Furthermore, we here propose a new power-law scaling which is applicable to other levels including 2p, 2s, and so on as well as 1s level, which is different from the power-law scaling in the previous study^S15^.

Here, we define $E_{c}=-e^{2}\left\langle R^{-1} \right\rangle_{n}/4\pi\varepsilon$, and $E_{k}=\hbar^{2}{\left\langle R^{-1} \right\rangle_{n}}^{2}/2m$, where $R$ is replaced with ${\left\langle R^{-1} \right\rangle_{n}}^{-1}$, for simplicity of calculation. Since $\left\langle R^{-1} \right\rangle_{n}$ only depends on the principal quantum number $n$ as

$$\begin{aligned} \left\langle R^{-1} \right\rangle_{n}=\frac{1}{a_{B}n^{2}}, \#\left( S7 \right) \end{aligned}$$

where $a_{B}=4\pi\varepsilon\hbar^{2}/me^{2}$ is the Bohr radius^S16^, we obtain

$$\begin{aligned} E_{c}=-\frac{2Ry}{n^{2}}, \#\left( S8 \right) \end{aligned}$$

and

$$\begin{aligned} E_{k}=\frac{Ry}{n^{4}}. \#\left( S9 \right) \end{aligned}$$

Equations (S6-S9) lead to

$$\begin{aligned} \frac{\left| E_{b} \right|}{E_{k}}=n^{2}, \#\left( S10 \right) \end{aligned}$$

and

$$\begin{aligned} \frac{\left| E_{c} \right|}{E_{k}}=2n^{2}. \#\left( S11 \right) \end{aligned}$$

Figure S8(a) plots these values for $n=1, 2, 3$, which follow $\left| E_{b} \right|/E_{k}=C\left( \left| E_{c} \right|/E_{k} \right)^{\beta}$, where $\beta=1$ and $C=0.5$. The order of exciton levels is the opposite of that of $E_{b}$ due to $E_{k}$ in the denominator.


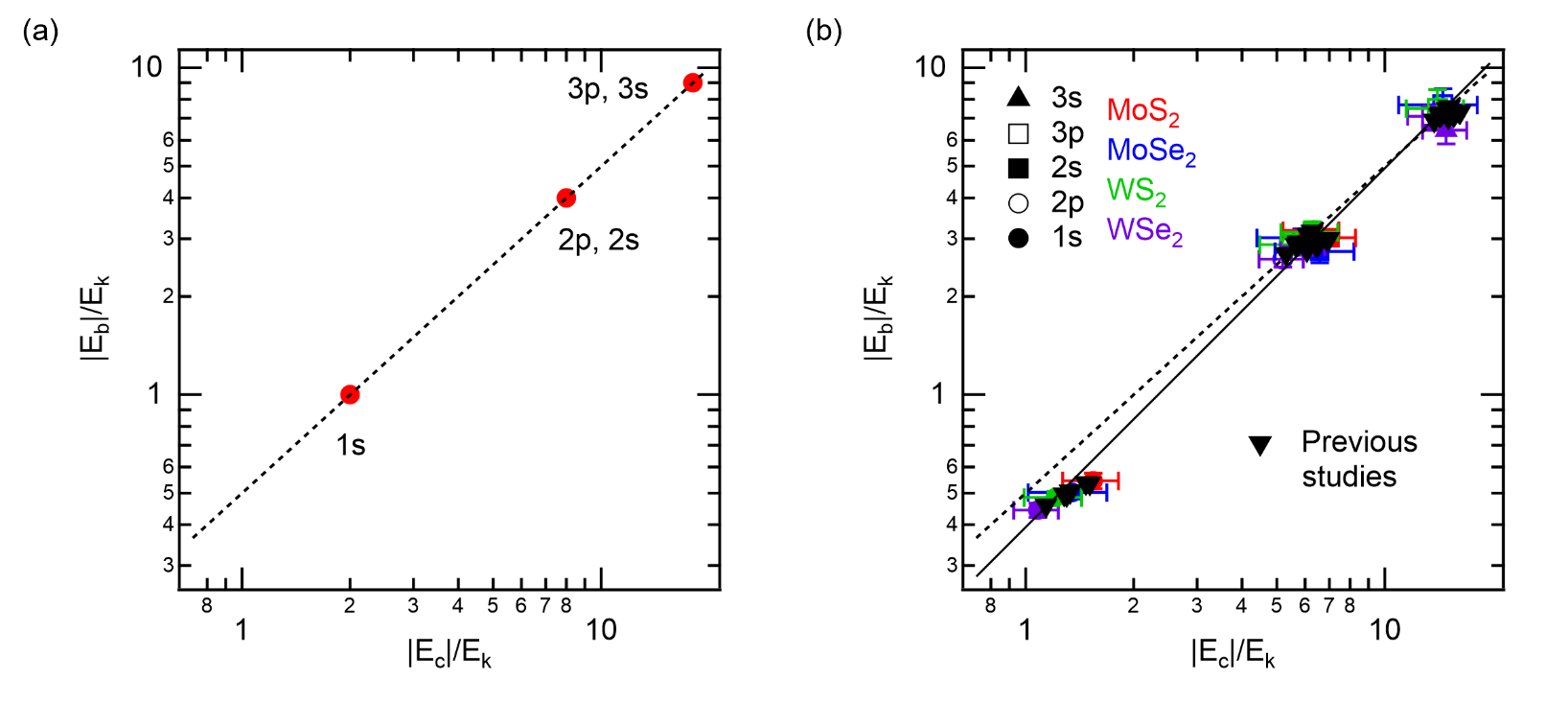


FIG. S8. Absolute values of the scaled binding energy $\left| E_{b} \right|/E_{k}$ plotted against Coulomb potential energy $\left| E_{c} \right|/E_{k}$, where $E_{k}$ is kinetic energy for (a) hydrogen model and (b) 1L-TMDs with the RKP model, which is the same as Fig. 4 of the main text. In Fig. S8(b), calculated results with parameters adopted from previous studies^S7-S10, S12^ are also shown with up to $n=3$ by down triangles.

# S9. Power-law scaling for 1L-TMD excitons

We calculate $E_{b}$, $E_{c}$, and $E_{k}$ for 1L-TMD excitons similarly to the 3D hydrogen model described in the previous section. $E_{b}$ of each exciton level is calculated by the experimentally obtained exciton energy minus the numerically obtained band-gap energy $E_{g}$. $E_{c}$ and $E_{k}$ are calculated as

$$\begin{aligned} E_{c}=-\frac{e^{2}}{4\pi\varepsilon_{0}\kappa}\left\langle R^{-1} \right\rangle_{nl},\#\left( S12 \right) \end{aligned}$$

and

$$\begin{aligned} E_{k}=\frac{\hbar^{2}}{2m}{\left\langle R^{-1} \right\rangle_{nl}}^{2}, \#\left( S13 \right) \end{aligned}$$

where $\left\langle R^{-1} \right\rangle_{nl}$ depends on the quantum numbers $n$ and $l$. The calculation procedure of $\left\langle R^{-1} \right\rangle_{nl}$ is described in the next paragraph of the section. Figure S8(b) plots the results, which is compared with those for the 3D hydrogen model in Fig. S8(a). The order of exciton levels is again the opposite of that of $E_{b}$ due to $E_{k}$ in the denominator. We can see the similarity and difference between these; remarkably, the data for the energetically separated 2p and 2s levels are at almost the same position in the graph. In addition, we can see that all the data are well reproduced by the best fitted curve to the expression

$$\begin{aligned} \frac{|E_{b}|}{E_{k}}=C\left( \frac{|E_{c}|}{E_{k}} \right)^{\beta} \#\left( S14 \right) \end{aligned}$$

with $C=0.4\pm0.1$ and $\beta=1.1\pm0.1$ (solid line). These values coincide with that for the 3D hydrogen model within the errors. On the other hand, the data for the 1s levels in 1L-TMDs are found to be deviated slightly from the one for the 3D hydrogen model, although the data for the other levels such as 2p and 2s are well reproduced by the one for the 3D hydrogen model (dashed line). Moreover, the data for the 1s levels are distributed depending on the material.

The expected value of the reciprocal of the radius $\left\langle R^{-1} \right\rangle_{nl}$ is calculated by using the exciton wave functions $\Psi_{j}\left( \vec{r} \right)$, which are also obtained by calculating exciton binding energies, as ($j$ is the label for exciton level)

$$\begin{aligned} \left\langle R_{j}^{-1} \right\rangle=\Sigma_{\vec{r}}\frac{1}{r} \left| \Psi_{j}\left( \vec{r} \right) \right|^{2}. \#\left( S15 \right) \end{aligned}$$

In polar coordinate system, $1/r$ is canceled out with $r$, which comes from Jacobian, and it is possible to evaluate eq. (S15) numerically. This is not the case for $\langle R^{-2}\rangle$, which is another reason that we adopt $\left\langle R^{-1} \right\rangle^{2}$ in the calculation of $E_{k}$. The calculation results of eq. (S15) are summarized in Table S7. These values are compared in Fig. S9 with the expectations of the 3D hydrogen model in eq. (S7). The data for the levels other than the 1s are reproduced fairly well by $\left\langle R^{-1} \right\rangle_{\mathrm{RKP}}=\left\langle R^{-1} \right\rangle_{\mathrm{hydrogen}}$. For the 1s excitons, dielectric screening gets larger due to contributions of a 1L-TMD. This leads to smaller binding energy, and thus larger reciprocal of the radius.


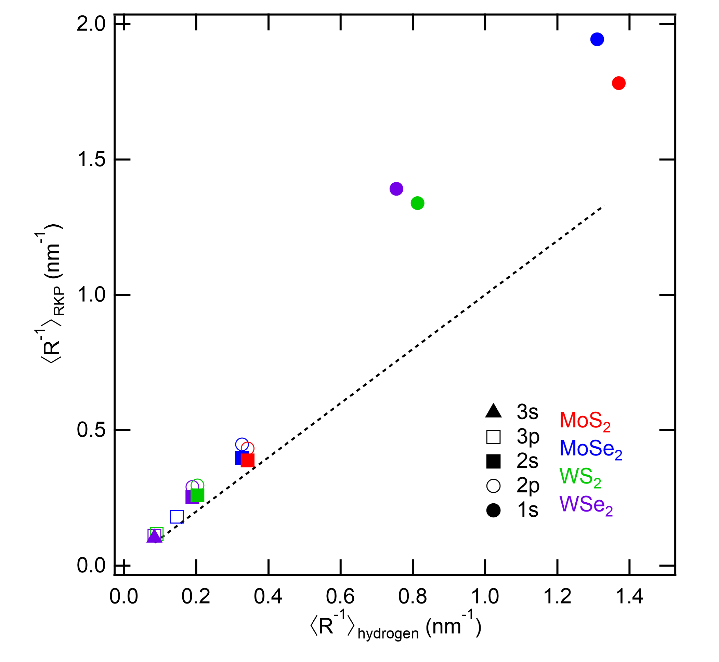


FIG. S9. Comparison of average reciprocal radius calculated with RKP model $\left\langle R^{-1} \right\rangle_{\mathrm{RKP}}$ in eq. (S15) and hydrogen model $\left\langle R^{-1} \right\rangle_{\mathrm{hydrogen}}$ in eq. (S7). The dashed line is $\left\langle R^{-1} \right\rangle_{\mathrm{RKP}}=\left\langle R^{-1} \right\rangle_{\mathrm{hydrogen}}$.

| Orbital | Expected value of the reciprocal of the radius $\langle R_{j}^{-1}\rangle$ (nm^-1^) | | | |
| --- | --- | --- | --- | --- |
|  | MoS_2_ | MoSe_2_ | WS_2_ | WSe_2_ |
| 1s | 1.78 | 1.94 | 1.34 | 1.39 |
| 2p | 0.432 | 0.448 | 0.296 | 0.290 |
| 2s | 0.388 | 0.398 | 0.260 | 0.254 |
| 3p | 0.180 | 0.181 | 0.116 | 0.111 |
| 3s | 0.167 | 0.167 | 0.107 | 0.102 |

Table S7. Expected value of the reciprocal of the radius $\langle R_{j}^{-1}\rangle$ calculated with the RKP model.

To examine that the differences of the obtained binding energies from the reported values do not influence our conclusion including the power law scaling in Fig. 4 of the main text, we calculate exciton binding energies using parameters of $m$, $\kappa$, and $r_{0}$ adopted from previous studies^S7–S10, S12^, which are listed in the second to the forth columns of Table S8. Calculated values of 1s exciton binding energy $|E_{b}^{1s}|$ are tabulated in the fifth column of Table S8, which are compared with the reported values in the previous studies in the sixth column. The results of the power-law scaling similar to Fig. 4 in the main text are shown by down triangle in Fig. S8(b) with up to $n=3$. These results are in line with our results with the errors of <~10% due to the normalization by $E_{k}$, because the errors of $E_{b}$, $E_{c}$, or $E_{k}$ themselves are ~20-30% at maxima. This consistency supports our conclusion of the power-law scaling.

| Material | $m$ ($m_{0}$) | $\kappa$ | $r_{0}$ (nm) | $\vert E_{b}^{1s}\vert$ (meV) | |
| --- | --- | --- | --- | --- | --- |
|  |  |  |  | Calculated in this study by using parameters | Reported in previous studies |
| MoS_2_ | 0.275 | 3.80 | 3.85 | 239.5 | - |
| MoSe_2_ | 0.35 | 5.05 | 3.82 | 207 | - |
| WS_2_ | 0.175 | 4.07 | 3.60 | 189 | - |
| WSe_2_ | 0.2 | 5.01 | 3.45 | 164 | - |
| MoS_2_^S12^ | 0.275 | 4.45 | 3.4 | 220.5 | 221 |
| MoSe_2_^S12^ | 0.35 | 4.4 | 3.9 | 232.3 | 231 |
| WS_2_^S12^ | 0.175 | 4.35 | 3.4 | 178.9 | 180 |
| WSe_2_^S7^ | 0.2 | 4.5 | 4.5 | 161.6 | 167 |
| WSe_2_^S8^ | 0.22 | 3.97 | 5.0 | 172.5 | 172 |
| WSe_2_^S9^ | 0.2 | 4.5 | 4.51 | 169.2 | 170 |
| WSe_2_^S10^ | 0.2 | 4.3 | 4.5 | 169.0 | 168.6 |

Table S8. (The third to sixth rows) Parameters used in calculations and calculated 1s binding energies $E_{b}^{1s}$ in this study, which are the same as in Table 2 in the main text. $m$ is the exciton reduced mass in units of free electron mass $m_{0}$, $\kappa$ the relative dielectric constant of hBN, and $r_{0}$ the screening length of 1L-TMD. (The other rows) Parameters adopted from previous studies and $E_{b}^{1s}$ calculated in this study by using the parameters. The values of $E_{b}^{1s}$ reported in the previous studies are also shown for comparison in the rightest column. The values of $E_{b}^{1s}$ for WSe_2_ are the same as in the second column of Table S6.

# S10. The asymptotic forms of the RKP and virial theorem

To understand the difference in the power-law scaling for 1s and other levels, we calculate the asymptotic forms of the RKP and virial theorem. First, we briefly review virial theorem referring to the textbook^S16^. Virial theorem insists that the following equation holds between potential energy $V$ and kinetic energy $T$;

$$\begin{aligned} 2\left\langle T \right\rangle=\left\langle\boldsymbol{r}\cdot\boldsymbol{\nabla}V \right\rangle, \#\left( S16 \right) \end{aligned}$$

where $\boldsymbol{r}$ is position operator. Particularly for centrifugal potential, eq. (S16) can be rewritten as

$$\begin{aligned} 2\left\langle T \right\rangle=\left\langle r\frac{dV}{dr} \right\rangle. \#\left( S17 \right) \end{aligned}$$

Then, we discuss the case for the RKP

$$\begin{aligned} V_{\mathrm{RK}}\left( r \right)=-\frac{e^{2}}{8\varepsilon_{0}r_{0}}\left[ H_{0}\left( \frac{\kappa r}{r_{0}} \right)-Y_{0}\left( \frac{\kappa r}{r_{0}} \right) \right].\#\left( S18 \right) \end{aligned}$$

This can be approximated for two extreme cases: For $\kappa r/r_{0}\gg1$ ^S17, S18^,

$$\begin{aligned} V_{\mathrm{RK}}\left( r \right)\sim V_{>}\left( r \right)=-\frac{e^{2}}{4\pi\varepsilon_{0}\kappa r},\#\left( S19 \right) \end{aligned}$$

which can be used for exciton levels with large radii. For $\kappa r/r_{0}\ll1$ ^S18, S19^,

$$\begin{aligned} V_{\mathrm{RK}}\left( r \right)\sim V_{<}\left( r \right)=\frac{e^{2}}{4\pi\varepsilon_{0}r_{0}}\left[ \ln\left( \frac{\kappa r}{2r_{0}} \right)+\gamma\right], \#\left( S20 \right) \end{aligned}$$

where $\gamma$ is Euler’s gamma. This can be used for exciton levels with small radii. By plugging eqs. (S19) and (S20) into eq. (S17) and given that the expectation value of total Hamiltonian is nothing but binding energy, one can obtain the relation between the binding energy and the expectation value of potential energy: For $\kappa r/r_{0}\gg1$,

$$\begin{aligned} E_{b}=\frac{1}{2}\left\langle V_{>} \right\rangle, \#\left( S21 \right) \end{aligned}$$

and for $\kappa r/r_{0}\ll1$,

$$\begin{aligned} E_{b}=\left\langle V_{<} \right\rangle+\mathrm{const}., \#\left( S22 \right) \end{aligned}$$

where the constant term in eq. (S22) is $e^{2}/8\pi\varepsilon_{0}r_{0}$. Equation (S21) is nothing but the power-law scaling for the 3D hydrogen (the dotted line in Fig. 4 and Figs. S8(a-b)) which reproduces well the behavior of levels other than 1s. Equation (S22) indicates that deviations from the power-law scaling for the 3D hydrogen model occur for exciton level with smaller radii. For 1s excitons, $\kappa r/r_{0}\sim1$, which is located in the intermediate of the two limits. That is why the deviations from the 3D hydrogen model are observed for 1s excitons in Fig. 4. In the intermediate case, the RKP cannot be approximated by a simple form, which makes it difficult to discuss the power-law scaling of 1s exciton only.

**References for supplementary information**

S1. Jin, C. *et al.* Interlayer electron-phonon coupling in WSe_2_/hBN heterostructures. *Nat. Phys.* **13**, 127–131 (2017).

S2. Markeev, P. A. *et al.* Energy-level alignment at interfaces between transition-metal dichalcogenide monolayers and metal electrodes studied with Kelvin probe force microscopy. *J. Phys. Chem. C* **125**, 13551–13559 (2021).

S3. Kusaba, S. *et al.* Broadband sum frequency generation spectroscopy of dark exciton states in hBN-encapsulated monolayer WSe_2_. *Opt. Express* **29**, 24629-24645 (2021).

S4. Liu, E. *et al.* Exciton-polaron Rydberg states in monolayer MoSe_2_ and WSe_2_. *Nat. Commun.* **12**, 6131 (2021).

S5. Li, Z. *et al.* Revealing the biexciton and trion-exciton complexes in BN encapsulated WSe_2_. *Nat. Commun.* **9**, 3719 (2018).

S6. Paur, M. *et al.* Electroluminescence from multi-particle exciton complexes in transition metal dichalcogenide semiconductors. *Nat. Commun.* **10**, 1709 (2019).

S7. Stier, A. V. *et al.* Magnetooptics of exciton Rydberg states in a monolayer semiconductor. *Phys. Rev. Lett.* **120**, 057405 (2018).

S8. Liu, E. *et al.* Magnetophotoluminescence of exciton Rydberg states in monolayer WSe_2_. *Phys. Rev. B* **99**, 205420 (2019).

S9. Chen, S.-Y. *et al.* Luminescent emission of excited Rydberg excitons from monolayer WSe_2_. *Nano Lett.* **19**, 2464–2471 (2019).

S10. Wang, T. *et al.* Giant valley-polarized Rydberg excitons in monolayer WSe_2_ revealed by magneto-photocurrent spectroscopy. *Nano Lett.* **20**, 7635–7641 (2020).

S11. Raja, A. *et al.* Dielectric disorder in two-dimensional materials. *Nat. Nanotechnol.* **14**, 832–837 (2019).

S12. Goryca, M. *et al.* Revealing exciton masses and dielectric properties of monolayer semiconductors with high magnetic fields. *Nat. Commun.* **10**, 4172 (2019).

S13. Slobodeniuk, A. O. & Molas, M. R. Exciton spectrum in atomically thin monolayers: The role of hBN encapsulation. *Phys. Rev. B* **108**, 035427 (2023).

S14. Geick, R., Perry, C. H. & Rupprecht, G. Normal modes in hexagonal boron nitride. *Phys. Rev.* **146**, 543–547 (1966).

S15. Perebeinos, V., Tersoff, J. & Avouris, P. Scaling of excitons in carbon nanotubes. *Phys. Rev. Lett.* **92**, 257402 (2004).

S16. Schiff, L. I. *Quantum Mechanics* (3rd Edition, International Student Edition) (McGraw-Hill, 1955).

S17. Rytova, N. S. The screened potential of a point charge in a thin film. *Moscow Univ. Phys. Bull.* **3**, 30 (1967).

S18. Keldysh, L. V. Coulomb interaction in thin semiconductor and semimetal films. *JETP Lett.* **29**, 658 (1979).

S19. Prada, E., Alvarez, J. V., Narasimha-Acharya, K. L., Bailen, F. J. & Palacios, J. J. Effective-mass theory for the anisotropic exciton in two-dimensional crystals: Application to phosphorene. *Phys. Rev. B* **91**, 245421 (2015).
